# Flexible Organic Crystalline Fibers and Loops with Strong Second Harmonic Generation

**DOI:** 10.1021/jacs.5c00598

**Published:** 2025-03-20

**Authors:** Jiawei Lin, Shi Tang, Liang Li, Liwen Fang, Qun Zeng, Guangxu Sun, Songgu Wu, Panče Naumov, Junbo Gong

**Affiliations:** † School of Chemical Engineering and Technology, State Key Laboratory of Chemical Engineering, Tianjin University, Tianjin 300072, China; ‡ Haihe Laboratory of Sustainable Chemical Transformations, Tianjin 300192, China; § Smart Materials Lab, New York University Abu Dhabi, P.O. Box 129188, Abu Dhabi, UAE; ∥ Novel Materials Development Lab, Sorbonne University Abu Dhabi, P.O. Box 38044, Abu Dhabi, UAE; ⊥ XtalPi Inc., Shenzhen Jingtai Technology Co., Ltd., Shenzhen 518100, China; # Center for Smart Engineering Materials, New York University Abu Dhabi, P.O. Box 129188, Abu Dhabi, UAE; ∇ Research Center for Environment and Materials, Macedonian Academy of Sciences and Arts, Bul. Krste Misirkov 2, MK-1000 Skopje, Macedonia; ○ Molecular Design Institute, Department of Chemistry, New York University, 100 Washington Square East, New York, New York 10003, United States

## Abstract

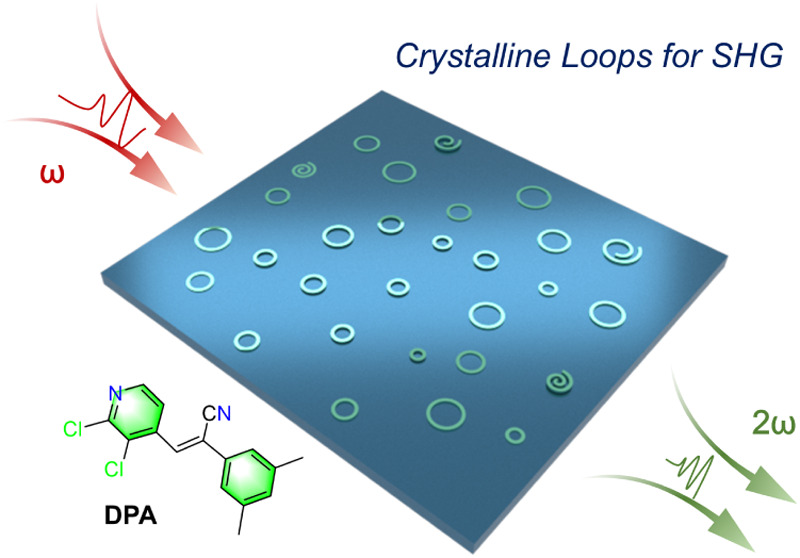

Flexible organic crystals represent a novel class of smart materials that open many opportunities for optical applications. While it has been established that elastic or plastic deformation of slender molecular crystals can be commonly induced by external intervention, crystals that grow in bent or curled shapes naturally are rarely reported. This study introduces an extraordinarily flexible organic crystalline fibrous material, (*Z*)-3-(2,3-dichloropyridin-4-yl)-2-(3,5-dimethylphenyl)acrylonitrile (DPA), that crystallizes both as straight and curled crystals. Crystals of DPA are easily obtained from solution either as long fibers or as crystals that are curled to various extent, and sometimes even closed into a loop. The straight crystalline fibers can be bent mechanically by applying force or photochemically by exposure to ultraviolet light. The straight and curled crystals are both polar and capable of highly efficient second harmonic generation (SHG) with respective intensities of 2.03 ± 0.15 and 1.52 ± 0.12 times (equivalent strain ≈ 1%) that of urea. Curling during crystal growth provides direct access to curved SHG-active flexible organic optical waveguiding elements, such as crystalline optical ring resonators, thereby circumventing the necessity for manual crystal bending, which is usually not readily scalable. This work highlights the unconventional properties and capabilities that fibrous molecular crystalline materials bring to the global materials space and their potential applications as shape-conforming, nonlinear organic materials.

## Introduction

**Figure 1:**
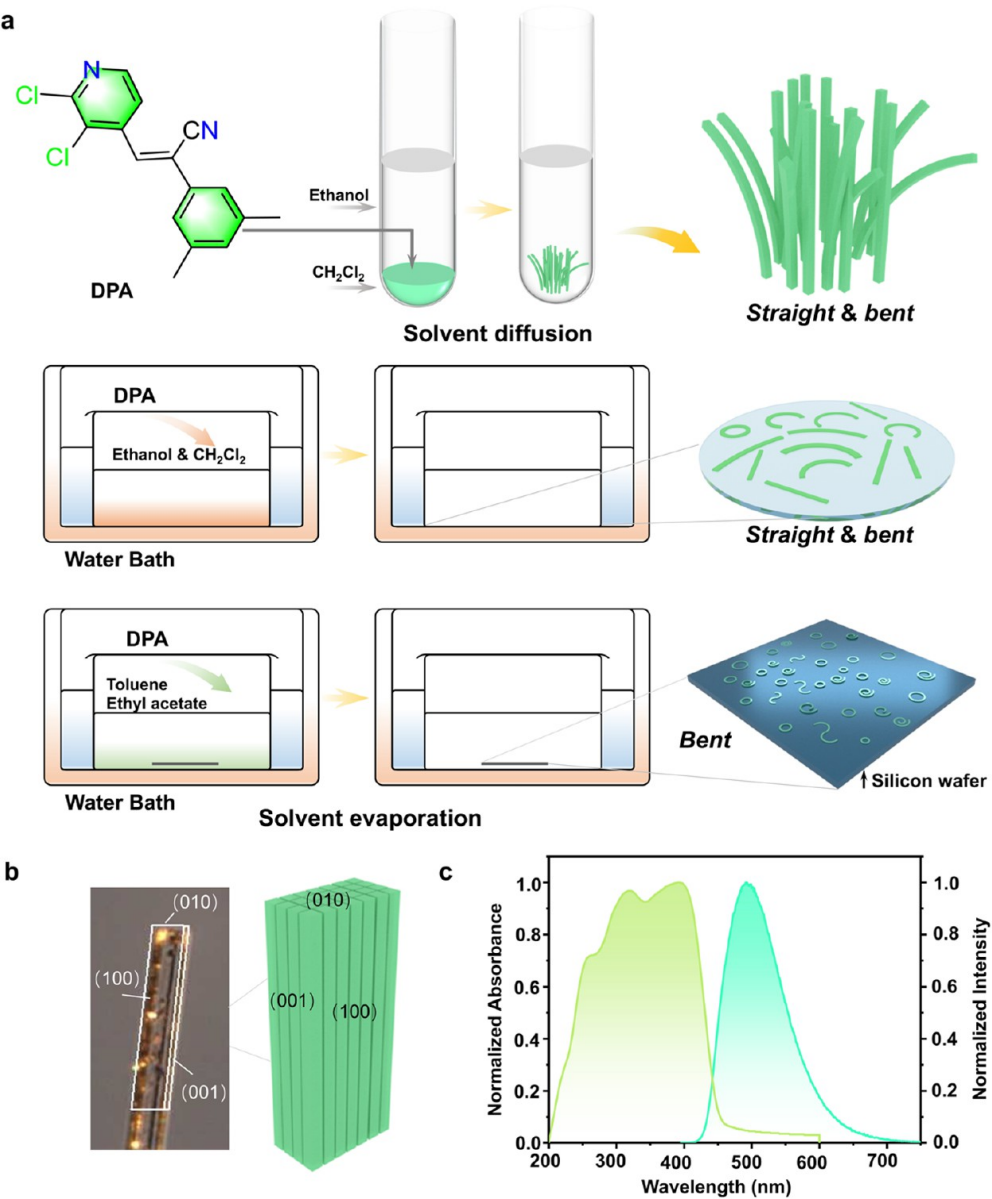
Preparation, face indexing, and electronic spectra of straight and curled crystals of DPA. (a) Chemical structure of DPA and schematic representation of the methods used for the preparation of crystals with two distinct habits. (b) Face indexing of a straight crystal of DPA. (c) Room-temperature UV–vis absorption and fluorescence spectra of solid DPA (the latter was obtained by excitation at 365 nm).

With the advancement of solid-state chemistry and crystal engineering, it has become evident that some ordered solids, such as the molecular crystals, can be unexpectedly dynamic and mechanically compliant.^1−3^ Such crystals become adaptive and can respond when affected externally, for example, by bending,^4,5^ twisting,^6−8^ or coiling.^9^ Crystals that can be reshaped, propelled, or even disintegrated by using light are particularly interesting due to their remotely controllable shapes, and consequently their optical, electrical, and other properties.^2,10,11^ Modulation of their properties upon deformation opens up unique opportunities for fabrication of compositionally versatile optical waveguides,^12,13^ flexible organic semiconductors,^14,15^ and numerous other deformable optoelectronic components.^16,17^ From the crystals that are known to respond to light, photochemically reactive *dynamic* crystals have recently emerged as an exciting new subcategory of rapid light-to-mechanical energy transducers.^3,18^ The materials chemistry community has developed multiple approaches to achieve the conversion of energy through photoresponsive mechanisms, with the key strategies including *trans–cis* photoisomerization,^19^ photocycloaddition,^20,21^ and photocyclization.^22^ Among these, the intermolecular [2 + 2] photocycloaddition exhibits well-characterized reaction pathways,^20,23^ with their structural prerequisites and stereochemical outcomes being governed by established geometric principles—most notably, the Schmidt’s criteria for photodimerization.^24^ Unlike most crystals that are obtained in regular geometric shapes, when confronted by physical obstacles or upon incorporation of defects during growth, certain compounds are known to separate from solution as crystals of peculiar shapes, such as arcs^16,25,26^ or helices.^27,28^ From melt, crystals sometimes easily grow as twisted lamellae or fibers in banded spherulites,^29,30^ with the actual crystal shape depending on temperature, impurities, and other factors.^31^ This approach not only provides access to uncommon crystal structures but also implies that twisted crystals are more common than it is commonly thought.^28^ Though templates could be, at least in principle, utilized to induce bending of crystals during growth,^32,33^ preparation of bent single crystals via template-free approaches is known to be challenging. Some of us recently reported luminescent crystals that appear to adopt Möbius strip topology when grown from a mixture of acetonitrile and toluene,^26^ an unusual shape that could elicit exotic physical properties.^16,34,35^ Given the natural advantages of weak intermolecular interactions—such as π···π interactions, weak hydrogen bonding, and halogen bonding—in facilitating molecular movement,^1,4,7,10,11^ it can be surmised that crystals prone to deformation should have weaker interactions. The cyanostyrene derivatives are classical π-conjugated emissive molecules known for being synthetically very accessible and have been already demonstrated to crystallize as flexible crystals that respond to mechanical force, ultraviolet radiation, and even solvent.^27,36−38^ Here, we report that under specific conditions crystals of a cyanostyrene derivative, (*Z*)-3-(2,3-dichloropyridin-4-yl)-2-(3,5-dimethylphenyl)acrylonitrile (DPA, Figure 1a), can be prepared in both straight and curled geometries. DPA crystals are morphed into curved or even circular shapes both during growth and upon application of mechanical force. Unlike other curved crystals, this material displays prominent nonlinear optical properties, which are detected and quantified for both straight and circular crystals, setting the platform for the preparation of other materials for scalable, SHG-active, all-organic flexible optical devices.

## Results and Discussion

**Figure 2:**
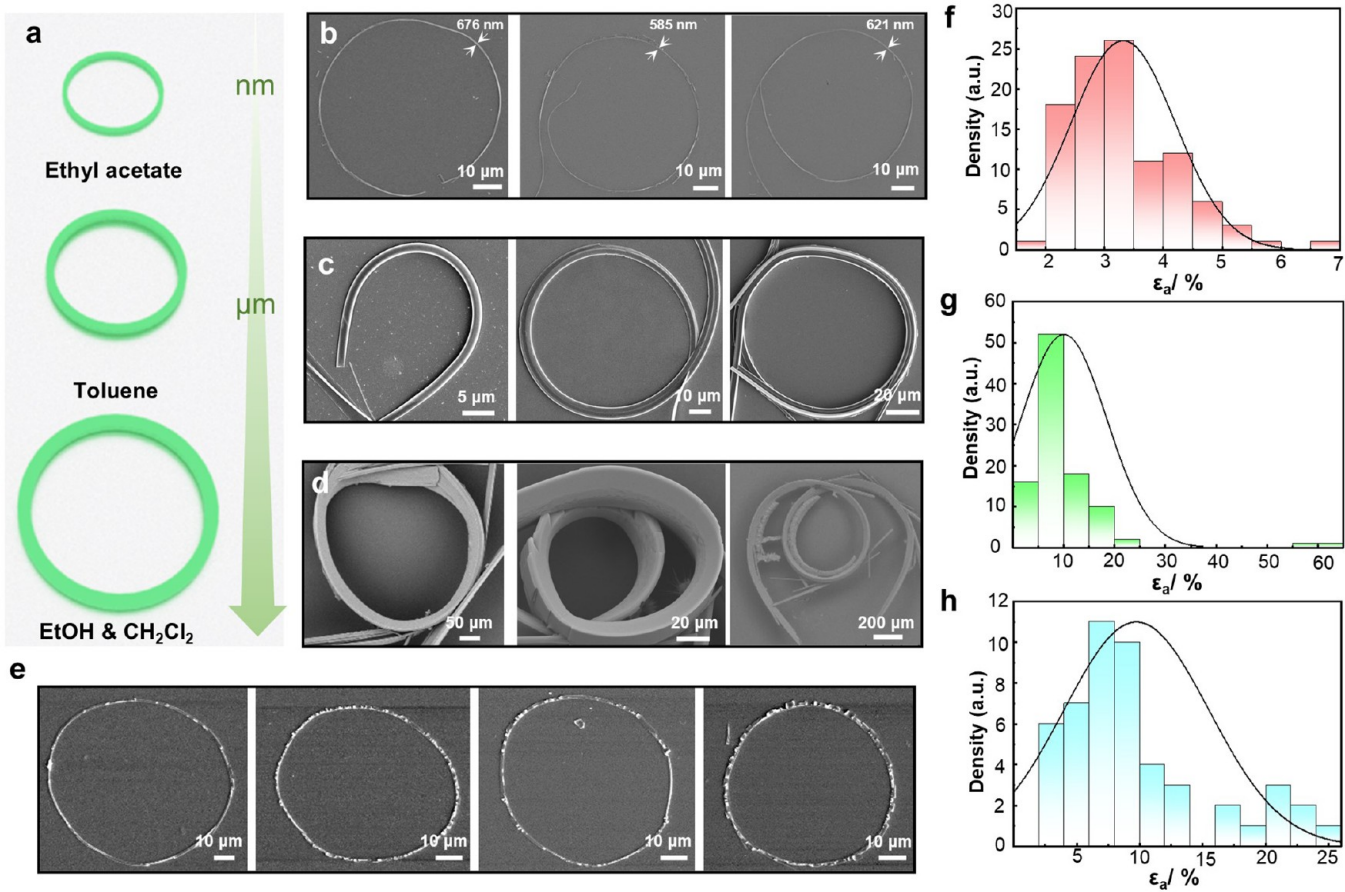
Analysis of the habits of DPA crystals grown from different solvents. (a) Schematic diagram showing the dependence of the size of the circular DPA crystals on the crystallization solvent. (b–d) SEM images of DPA obtained from ethyl acetate (b), toluene (c), and a mixture of ethanol and dichloromethane (d). (e) Crystal rings of DPA obtained from ethyl acetate. (f–h) “Equivalent strain” distribution of DPA crystals obtained from ethyl acetate (f), toluene (g), and a mixture of ethanol and dichloromethane (h).

DPA was prepared by condensation between 2-(3,5-dimethylphenyl)acetonitrile and 2,3-dichloroisonicotinaldehyde (Scheme S1 and Figure S1). Both solvent diffusion and solvent evaporation were used to grow the crystals (Figure 1a). Long needle-like crystals were obtained by layering ethanol on top of a dichloromethane solution of DPA and allowing for slow solvent diffusion at 5 °C (Figures 1a and S2). The face indexing of the as-obtained crystals showed that they had two sets of well-developed faces: a wide (100) face and a narrow (001) face (Figure 1b). Under UV light, the crystals absorbed mostly between 200 and 450 nm and exhibited strong green fluorescence with a maximum wavelength λ_max_ = 493 nm for excitation at 365 nm (Figures 1c, S3, and S4). Unexpectedly, however, some crystals prepared by solvent diffusion were obtained with a curled habit (Figures 2a and S2). The propensity for curling during growth was also examined by solvent evaporation from several other solvents, including ethyl acetate, toluene, and a mixture of dichloromethane and ethanol, at 25 °C (Figures 2b-d and S5). From ethyl acetate, the compound crystallized as strongly curled or even circular crystals with widths that typically ranged from hundreds of nanometers to several micrometers (Figures 2b and S5a). A significant portion of the crystals were obtained as rings although the loops were often notched. Nearly perfectly circular crystals and even completely closed loops were also found but were relatively rare compared to the other shapes. Scanning electron microscopy (SEM) revealed that the surfaces of these crystals are irregular (Figures 2e and S6).

Compared to the crystals obtained from ethyl acetate, the as-grown bent crystals were less prevalent in the batches obtained from toluene; the latter were also wider (typically, around 5 mm) and had smoother surfaces (Figures 2c and S5b). Crystals grown from a mixture of dichloromethane and ethanol, by both diffusion and evaporation, were the largest and were long up to several centimeters (Figures 2d, S5c, and S7). A few crystals were found to be closed into a circle and had satellite branches and defects (Figures 2d and S8). The micro-Raman spectra of the straight and curled crystals were consistent and confirmed that they were of the same composition and crystallographic phase (Figure S9). The difference in size is qualitatively attributed to the rate of crystallization depending on the solvent, and it may be related to its polarity.^39,40^

While most of the crystals were observed to be bent on their (001) facets, the bending preference was found to depend on the solvent used for crystallization. We determined the ratio of crystals depending on their bending plane and their “equivalent strain”—the strain to which they would be subjected if they were mechanically bent starting from a straight shape (note that the curled crystals are already in equilibrium, hence the use of the term “equivalent”; this term does not refer to actual physical strain). By analyzing about 100 crystals obtained from each solvent, we found that 95% of the crystals grew from ethyl acetate bent on (001). This ratio was lower, 89% and 74%, for crystals obtained from toluene and a mixture of dichloromethane and ethanol, respectively. Given the natural width of the crystal facets, at identical curvature, it is expected that a crystal would be subjected to higher strain when it is bent on (001) compared to bending on the (100) facet, based on the Euler–Bernoulli equation ε_*n*_ = *t*/(*d* + *t*),^41^ where *t* is the thickness of the crystal and *d* is the diameter of the inner arc. The observation that most crystals were still bent on the (001) facet indicates that relative to the mechanical bending, the bending during crystallization is predominantly governed by other factors. The “equivalent strain” of most crystals obtained in the bent state from ethyl acetate was less than 5% (Figure 2f). However, when the crystals were obtained from toluene, the values of the “equivalent strain” were mostly between 5 and 20%, with the strain of some crystals of up to 60% (Figure 2g). The strain of 28% crystals obtained from a mixture of ethanol and dichloromethane was above 10%, and for 12% crystals, it was greater than 20% (Figure 2h).

**Figure 3:**
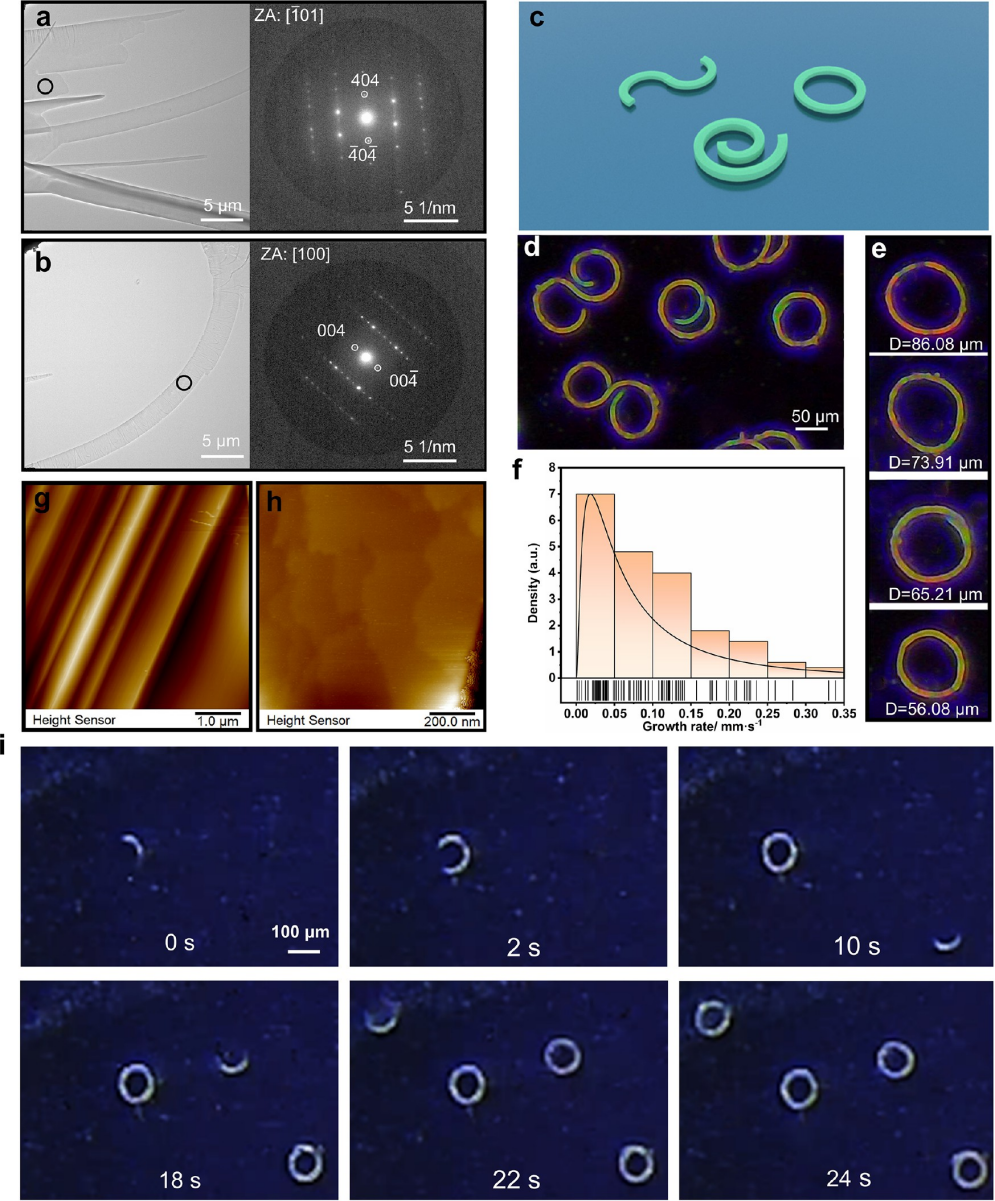
Bent DPA crystals grown from ethyl acetate. (a, b) Bright-field cryo-TEM images and the corresponding selected-area electron diffraction (SAED) patterns. (c) A schematic diagram of three typical habits of DPA. (d, e) Microscopic images of the DPA crystals. (f) Growth rate distribution of DPA when it crystallizes from ethyl acetate. (g, h) Topography, observed by atomic force microscopy, of the crystals grown from a mixture of ethanol and dichloromethane (g), and ethyl acetate (h). (i) Time evolution of the circular DPA crystals during their growth (the time stamps are shown on each panel).

The selected-area electron diffraction (SAED) patterns showed that straight crystals from ethyl acetate had sharp reflections and good crystallinity (Figure 3a). Unlike mechanically bent crystals, where significant loss of the long-range structural order is usually observed upon plastic bending,^42,43^ the circular DPA crystals had sharp diffraction peaks devoid of diffuse scattering (Figure 3b). Due to the small size of the crystalline rings from ethyl acetate, we were not able to determine their crystal structure by using X-ray diffraction; instead, the straight region of a slightly bent crystal obtained from a mixture of dichloromethane and ethanol was used for structure determination. It was confirmed that they have the same unit cell as the straight crystals (Table S1). As expected, the bending of the crystals visibly distorted the Bragg spots, similar to other cases of mechanically deformed crystals (Figure S10).^42,43^

The mechanism of crystal growth was studied by direct observation of the solvent evaporation process. Nonsaturated ethyl acetate solutions of different concentrations (0.5, 1.0, 1.5, 2.0, and 2.5 mg mL^–1^) were prepared and placed in 5 mL beakers. A silicon wafer was placed at the bottom of each beaker, and the beaker was covered with a glass plate and kept at 25 °C. Movies S1 and S2 and Figure S11 show that the wafers were covered with bent, belt-like crystals. The crystals developed into three typical shapes (Figure 3c–e), depending on the growth orientation of the two ends of the microbelt (Figure S12). The growth process of the crystalline loops is shown in Figure 3i. The bent shape did not depend on the nature of the substrate, and the same shape was obtained when crystals were grown on a glass substrate instead of silicon (the silicon substrate was convenient for SEM imaging). The growth rate was studied for concentrations between 0.5 and 2.5 mg mL^–1^ on a total of 100 bent crystals and was found to depend on the concentration of the solute (Figure S13). Faster growth rates resulted in a smaller number of bent crystals (Figure 3f). The optimal concentration was determined to be 1 mg mL^–1^, and it generated the largest ratio of bent crystals to those of other shapes. The growth rate ranged from 0.01 to 0.14 mm s^–1^, and for most crystals, it was between 0.02 and 0.04 mm s^–1^ (Figure S13b).

As mentioned above, DPA crystals were obtained with different sizes ranging from nanoscopic to macroscopic dimensions. Crystals obtained from ethyl acetate and toluene were smaller and therefore easier to grow with a bent shape. Intrinsic stresses are easily released by crystals of small size and result in deformation,^28,44^ which correlates with a specific growth rate. However, DPA crystals from the mixed solvents with a large size still grew and were also bent. Observation of the crystal growth showed that bent crystals prepared from the mixed solvents were obtained by two mechanisms. First, the DPA nucleated and developed as microseeds with a width up to about several tens of micrometers (Movie S3). The ends of the fibers then extended at different rates to develop into large macroscopic crystals. Alternatively, DPA crystals initially grew into long and bent fine fibers, which later grew in width and thickened (Movie S4). These crystals appeared as crystalline aggregates that were composed of many fine fibers (Figure S8). The crystals had a smooth surface around the central section, but consisted of fibrous satellite crystals closer to the ends. Atomic force microscopy (AFM) showed striations on the (100) face of the crystals, indicating lamination of the crystals obtained from the mixed solvent (Figure 3g). On the contrary, no apparent layers could be observed for the curved crystals obtained from ethyl acetate (Figure 3h). DPA crystals with large size relied on the release of the intrinsic stress of the fibers and were intergrowths of the fine fibers, akin to the well-known twisted crystal of benzamide form II.^28^

**Figure 4:**
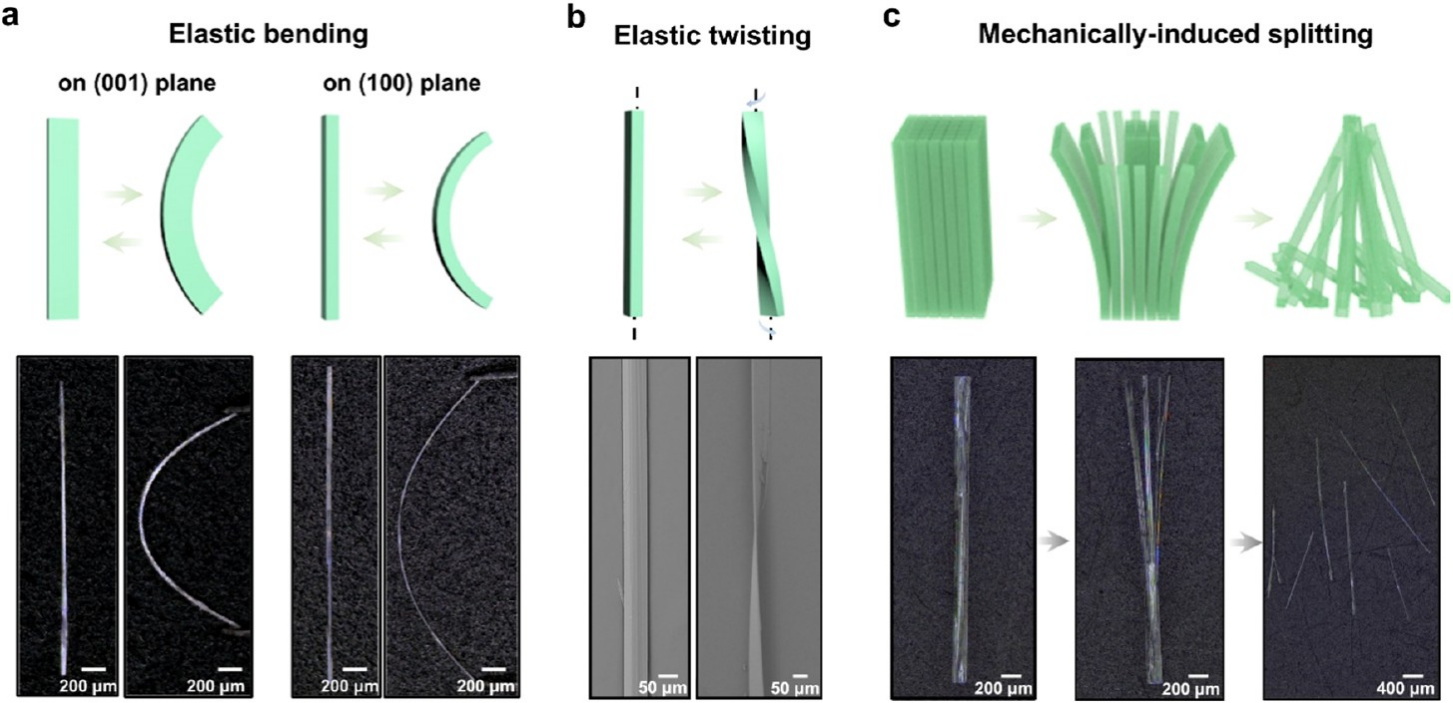
Mechanically induced deformation of DPA crystals: (a) Elastic bending along the (001) plane and (100) plane. (b) Elastic twisting. (c) Mechanically induced splitting.

Having the long and straight crystal habits obtained from solvent diffusion at hand, bending was attempted by applying mechanical force to evaluate their mechanical properties. When force was applied to the two ends of long needle-like crystals, they proved to be very flexible and bent along the (100) plane and (001) plane without fracturing (Figure 4a and Movie S5). The bent crystals reverted to their original straight configuration upon the removal of the applied force, demonstrating elasticity. This bending was repeated multiple times without any visible fracture or other changes in crystals’ appearance. The scanning electron micrographs showed that the bent planes remained smooth and without signs of delamination (Figure S14). Moreover, the flexibility on two facets, (100) and (001), turns DPA crystals susceptible to application of torque, and therefore, they can also be twisted (Figure 4b). The elastic strain of the most DPA crystals, calculated from 100 crystals, was around 1–3% and therefore similar to values reported for other cyanostilbene derivatives^36,37,45^ (Figure S15 and Table S2). Application of strain beyond the limit resulted in fracture, and the SEM images showed that this happens by partial delamination of the crystal into fibrillar lamellae that had separated along its long axis (Figure S16). However, the looplike crystals proved to be fragile under mechanical force. We hypothesize that their nonoptimal crystallinity accounts, among other factors, for their poor mechanical flexibility.

Nanoindentation was performed to further quantitatively assess the surface mechanical properties of the DPA crystals and to determine the elastic modulus and the hardness.^46^ The calculated average modulus was found to be 11.54 ± 0.45 GPa, and the hardness was 0.31 ± 0.02 GPa (Figure S17 and Table S3), values that are similar to other organic crystals^36−38,45^ (Table S4). Moreover, applying mechanical force on the (100) or (001) planes facilitated peeling of the DPA crystals into thinner, finer crystals (Figure 4c). The structure of the slender crystals was identical to that of the parent crystal (Table S1). These thinner crystals were also bendable on two faces, (100) and (001) (Figure S18), and the SEM images showed that their surfaces generated by fracture were smooth (Figure S19).

**Figure 5:**
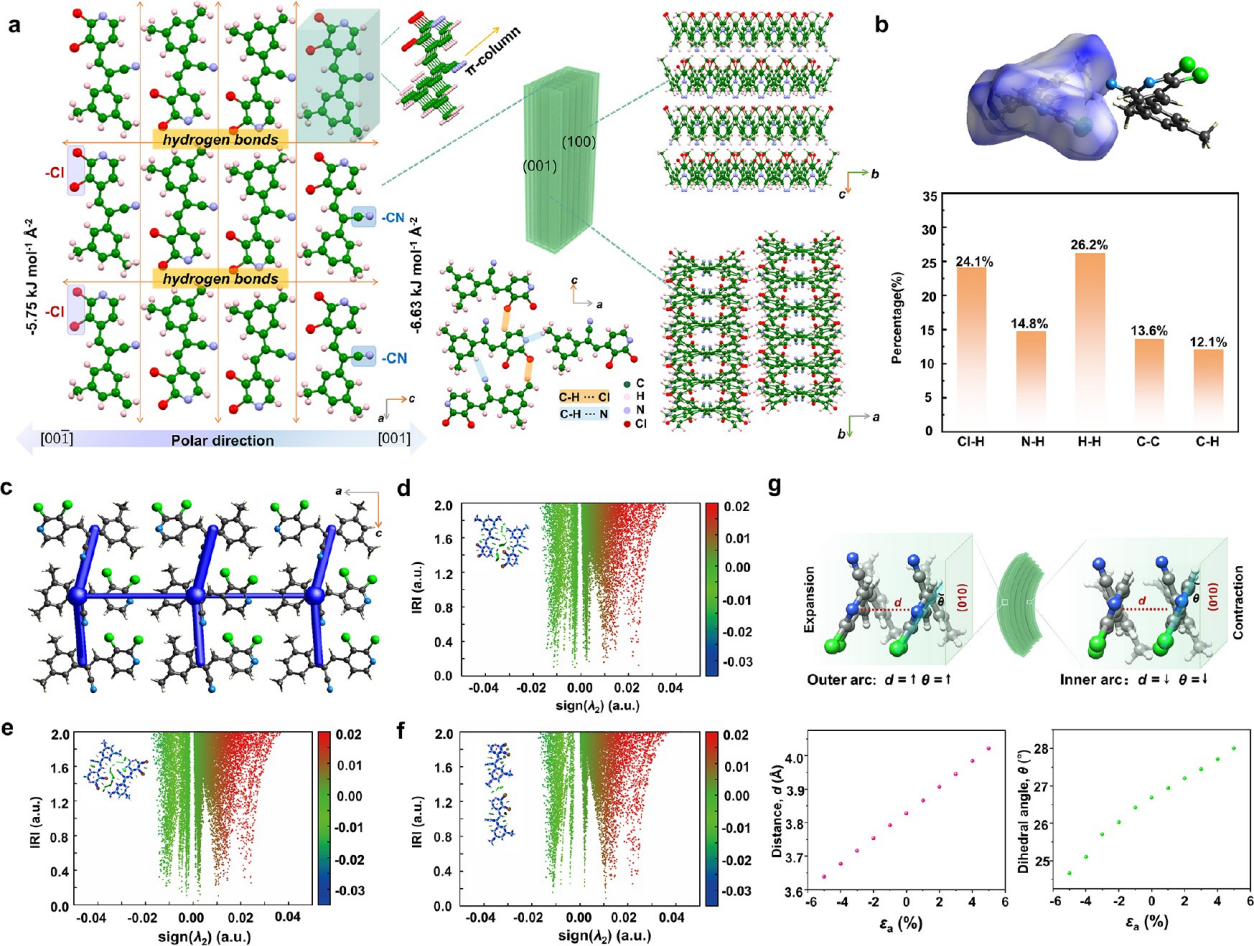
Proposed mechanism for the flexibility of DPA crystals. (a) Molecular packing in the crystal of DPA. (b) Hirshfeld surface analysis. (c) Energy frameworks for DPA along the *b* axis. (d–f) IRI (interaction region indicator) analysis for the typical noncovalent interactions. (g) The mechanism of elastic bending in the crystal of DPA. In these plots, *d* is the distance between the adjacent pyridine ring and θ is the dihedral angle between the pyridine ring and (010) plane.

In order to obtain some insights into the origins of the mechanical flexibility, we determined the crystal structure of DPA (CCDC number: 2346259). The crystals are orthorhombic, polar space group *Pca*2_1_, with unit cell parameters *a* = 25.3067(5) Å, *b* = 3.88190(10) Å, and *c* = 28.3415(6) Å, and have two molecules in the asymmetric unit (Table S1). The molecule of DPA adopts a nonplanar conformation, and the dihedral angles between the benzene and pyridine rings are 44.31/51.88° (Figure S20). The molecules interact with each other via π–π interactions (3.88 Å) and form π columns along the *b* axis. Along the *c* axis, the columns are aligned in parallel or antiparallel fashion, interacting via C–H···Cl (3.57 Å, 2.80 Å, 139.0°) in the parallel alignment and via C–H···N (3.58 Å, 2.71 Å, 151.7°) interactions and C–H···Cl (3.89 Å, 2.95 Å, 101.9°) interactions in the antiparallel one (Figure 5a). The pyridine nitrogen interacts with −CH_3_ via C–H···N (3.60 Å, 2.63 Å, 173.5°) interactions. It is noted that the molecules arrange themselves in a polar arrangement along the *c* axis (Figure 5a). The −CN groups are oriented along [001], while the -Cl groups are oriented along [001̅]. In the absence of detailed analysis, we hypothesize that the asymmetric packing arrangement provides structural inhomogeneity for bending during the crystal growth. The results appear to be consistent with density functional theory (DFT) calculations. The interface energy on (001) and (001̅) planes was calculated by using the software VASP.^47^ It showed that the surface energy changes were significantly different before and after optimization in vacuum: −6.63 kJ mol^–1^ Å^–2^ on the (001) plane and −5.75 kJ mol^–1^ Å^–2^ on the (001̅) plane, with a difference of 0.8 kJ mol^–1^ Å^–2^. It was found that the surface energy difference increased to 0.95 and 0.98 kJ mol^–1^ Å^–2^ in the case of ethyl acetate and toluene as solvents, respectively. We conclude that the solvation promotes structural adjustment during the crystal growth by differential adsorption on the (001) and (001̅) planes. As mentioned above, the bent crystals grown from a mixture of dichloromethane and ethanol exhibited larger equivalent strain and size than those from ethyl acetate and toluene. This result might be qualitatively related to the higher polarity of dichloromethane (Table S5),^39^ which indicates stronger interaction with the polar (5.51 D) DPA molecules. The calculated solvation-free energies confirmed the higher energy of solvation in the mixture of dichloromethane and ethanol (Table S5), although a more direct relation with the crystal habit would require consideration of multiple factors that become relevant at various stages of the nucleation and crystal growth process.

Visualization of the Hirshfeld surfaces showed that the distribution of H···H (26.2%) and Cl···H (24.1%) interactions has the highest share in the intermolecular interactions, revealing that the weak interactions were dominant (Figure 5b). Further quantification of the intermolecular interaction energies was performed based on the energy framework scheme^48,49^ (Figure 5c). Overall, the distribution of the interaction energy was anisotropic. Along the *b* axis, the total interaction energies of the π stacking columns were strongest, −55.1 kJ mol^–1^. The total energies of the slip planes along the *a* and *c* axes were −24.4 and −36.0 kJ mol^–1^, respectively. These low-energy slip planes could aid the structural rearrangement of the π-stacked molecular columns. Furthermore, the interaction region indicator (IRI) method^50^ was applied to analyze the three noncovalent interactions between the adjacent columns (Figure 5d–f). The color-coded sign(λ_2_)ρ function was mapped onto the isosurfaces to show the properties of the interaction region. In Figure 5d–f, the blue color represents the notable attraction (hydrogen bonding, halogen bonding), the green color indicates van der Waals (vdW) interactions, and the red color represents notable repulsive/steric interactions (Figure S21). All three synthons are related to the green region, showing weak vdW interactions (sign(λ_2_)ρ = [−0.02, 0.00]). These weak and dispersive interactions may be relevant to the structural adjustment in the crystal growth or elastic bending.

Some fundamental changes must occur in the shape of the crystal in order for a crystal to be elastically bent; the outer arc of the bent crystals should become longer while the inner arc is expected to become shorter.^51,52^ The uniaxial strain-energy calculations showed that the structural rearrangement could be facilitated by cooperative action between the expansion or constriction of the π-stacking interactions and partial molecular rotation (Figure 5g). Specifically, the distance between the adjacent pyridine ring along the *b* axis increases and the angle between the pyridine ring and the (010) plane also increases in the outer arc. In the inner arc, the distance and angle decrease. Along with the increasing strain, the structure perturbation increases in both the inner and outer arcs. These calculated changes qualitatively match well the molecular movement observed for an elastic deformation in a recent work using synchrotron microfocus X-ray diffraction by Clegg et al.^53^ The reversibility of this molecular movement is ascribed to the low energy cost related to the change in weak and dispersive interactions.

The preparation of new fibrous materials is an important development direction in materials science;^9,54,55^ however, most available natural fibrous materials are amorphous or partially crystalline. Several cases of elastic crystals have been reported that could be mechanically separated into thinner crystals.^36,56,57^ For example, mechanically induced crystal splitting similar to the one observed here was previously reported by Hayashi et al.^57^ with a compound based on π-conjugated, rigid, planar molecule. DPA molecules in the crystal exist in a nonplanar conformation. Our recent work showed that a nonaromatic organic molecular crystal capable of two-dimensional elastic bending can also be mechanically split into thinner crystals.^41^ The vertical stripes on the (100) plane of DPA were characterized by AFM and SEM (Figures S22 and S23). Furthermore, fine branches were clearly observed at the crystal ends. The crystals could be cut into fibers with a minimum width of about 10 μm by using tools (Figure S24). This unique surface morphology indicates that the propensity for mechanically induced splitting may be related to the mechanism of crystal growth. As mentioned above, the π-stacked columns interact with each other via C–H···Cl and C–H···N interactions in the orthogonal direction, and we expect that the mechanical splitting may involve disruption of these weak interactions.

**Figure 6:**
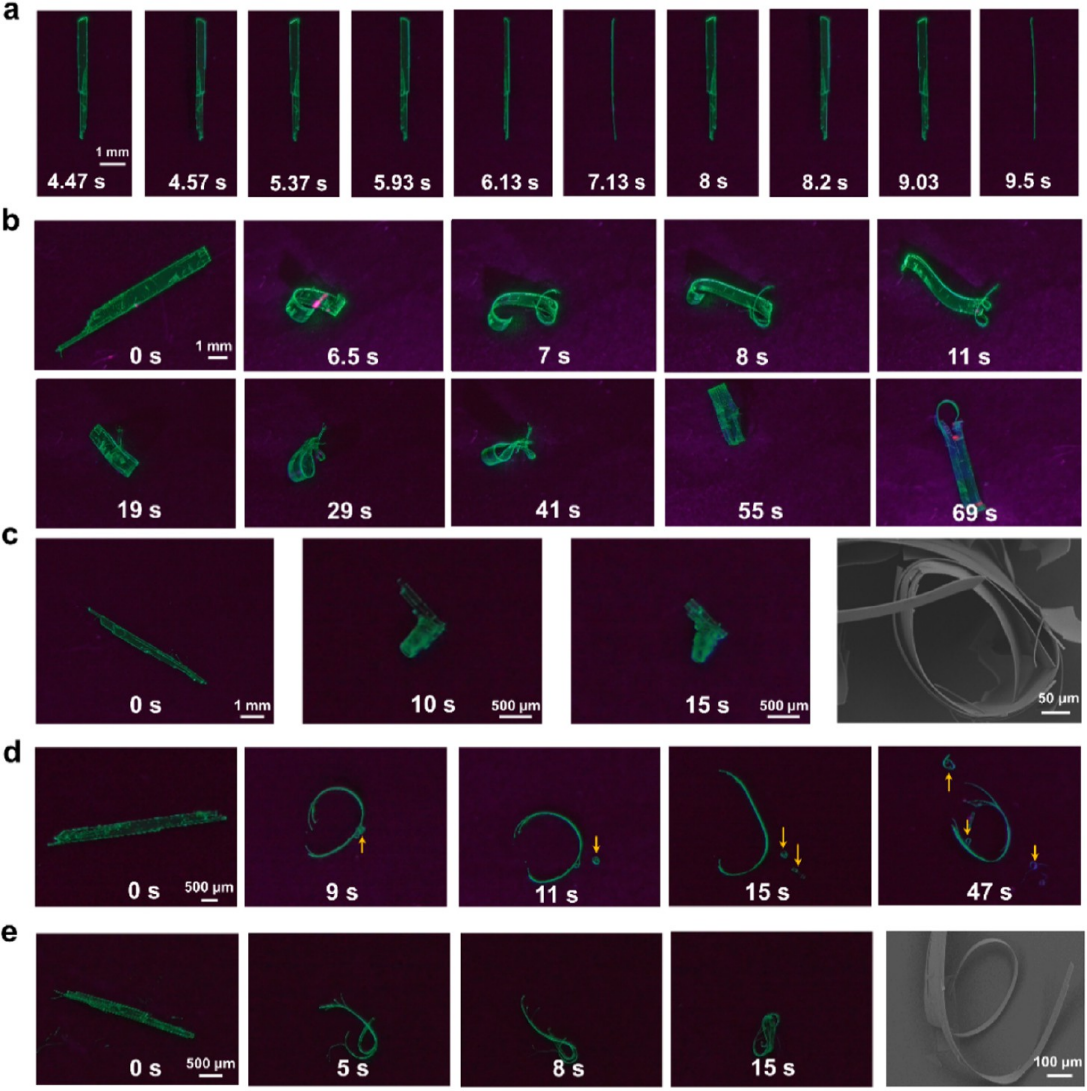
Photoresponsive behavior observed with DPA crystals: (a) flipping, (b) bending, (c) coiling, (d) splitting, and (e) peeling.

Given the presence of a double bond in the structure of DPA (Figure 1a), and similar to other unsaturated systems^58,59^ that are generally prone to dimerization under UV light and could result in photomechanical effects, we examined the behavior of DPA crystals upon exposure to UV radiation. The crystals were observed to respond by various mechanical effects including flipping, bending, coiling, splitting, and peeling. For instance, Figure 6a shows that a DPA crystal can flip up to 9 times in 5 s without obvious cracking. Thin crystals were particularly compliant and behaved similar to polymers; a thin crystal was easily bent under UV light and recovered its straight shape after the exposure to light was terminated (Figure 6b). Other crystals coiled into a pipe-like shape (Figure 6c). The deformation of some crystals was often accompanied by quick movements and even jumping, akin to the known photosalient effect.^60^ Apart from the deformation and motion, DPA crystals also exhibited disintegrative behavior, such as peeling and splitting, similar to the splitting induced by mechanical force (Figure 6d,e).

The kinetics and reversibility of the photoinduced bending were studied by lateral exposure of a slender crystal measuring 2.4 mm × 40 μm × 20 μm to UV light (broadband, main maximum at 365 nm) with an intensity of approximately 18 mW cm^–2^ (Figure S25). The bending rate decreased from an initial rate (expressed as the change of the bending angle) of 16 ± 2.4° s^–1^ soon after the onset of irradiation to 0° s^–1^ after 20 s of continuous exposure to light of the same crystal facet. Termination of the irradiation did not restore the original crystal shape due to the irreversibility of the [2 + 2] cycloaddition reaction; however, the crystal reverted to its straight shape by exposure to UV light from the opposite direction. The bending–unbending cycles without significant fatigue were performed 50 times to a bending angle of ∼4° and 30 times to an angle of ∼8°.

The course of the photochemical reaction that underlies these effects was monitored by ^1^H NMR spectroscopy (Figures S26 and S27). DPA powder was irradiated with UV light (365 nm), and samples were taken during the irradiation and dissolved in DMSO-*d*_6_ to record the ^1^H NMR spectra. New signals at 5.69 ppm emerged after 1 min of irradiation as evidence of the generation of a dimer, the substituted cyclobutane product 2,4-bis(2,3-dichloropyridin-4-yl)-1,3-bis(3,5-dimethylphenyl)cyclobutane-1,3-dicarbonitrile (DDC). The spectral analysis showed that 90% of DPA was converted to DDC within 30 min from the irradiation onset (Figure S27). The UV–vis absorption spectrum showed that DDC absorbs from 200 to 600 nm (Figure S28). The fluorescence spectrum corroborated this result with a distinct fluorescence peak of DPA around 493 nm, which contrasts the emission of pure DDC around 532 nm at an excitation wavelength of 405 nm (Figure S29). The [2 + 2] cycloaddition is possible due to the relative disposition of the reactive molecules that complies with the topochemical requirements for such a reaction, with *d* < 4.2 Å, θ_1_ = 0°, θ_2_ = 90°, and θ_3_ = 90° (for definition of these parameters, see Figure S30).^61,62^ In the structure of the reactant, two molecules of the “active dimers” (pairs of molecules amenable to dimerization) arrange in parallel and head-to-head manners, and as shown in Figure S31, there are two types of active dimers (types A and B). The distance between the adjacent C=C bonds in both dimer types is 3.88 Å. However, θ is slightly different in the adjacent active dimers due to the presence of two crystallographic types of molecules in the asymmetric unit: θ_1_ = 0°, while θ_2_ = 91.4°/91.0° and θ_3_ = 72.4°/76.0°. The pairs in the active dimers are favorably parallel, and the distance is <4.2 Å, thereby satisfying the Schmidt’s topochemical criteria for dimerization.^24^

Despite several attempts, direct determination of the structure of the dimer, DDC, in the crystal of the reactant was not feasible due to the detrimental impact of the reaction on the crystal structure. However, single crystals of DDC were obtained by recrystallization from a mixture of ethanol and dichloromethane, their structures were determined, and confirmed the chemical structure of the product of [2 + 2] photocycloaddition (Figure S32 and Table S1). In its pure form, DDC crystallizes in the monoclinic space group *P*2_1_/*c* with unit cell parameters *a* = 16.7285(5) Å, *b* = 23.4967(5) Å, *c* = 16.2061(4) Å, and β = 96.880(3)°, with two molecules in the asymmetric unit. However, the powder X-ray diffraction patterns of the samples before and after recrystallization were inconsistent with each other (Figure S33), showing that they have different crystal structures. Based on the geometric changes expected to occur upon dimerization, we hypothesized that the formation of DDC inevitably forces the pyridine and benzene rings to move away from the four-membered ring. Similar to other cases, steric incompatibility normally accounts for the occurrence of macroscopic mechanical effects, where a combination of change in volume, generation of defects, accumulation and release of lattice strain, and related processes results in a variety of kinematic effects.

**Figure 7:**
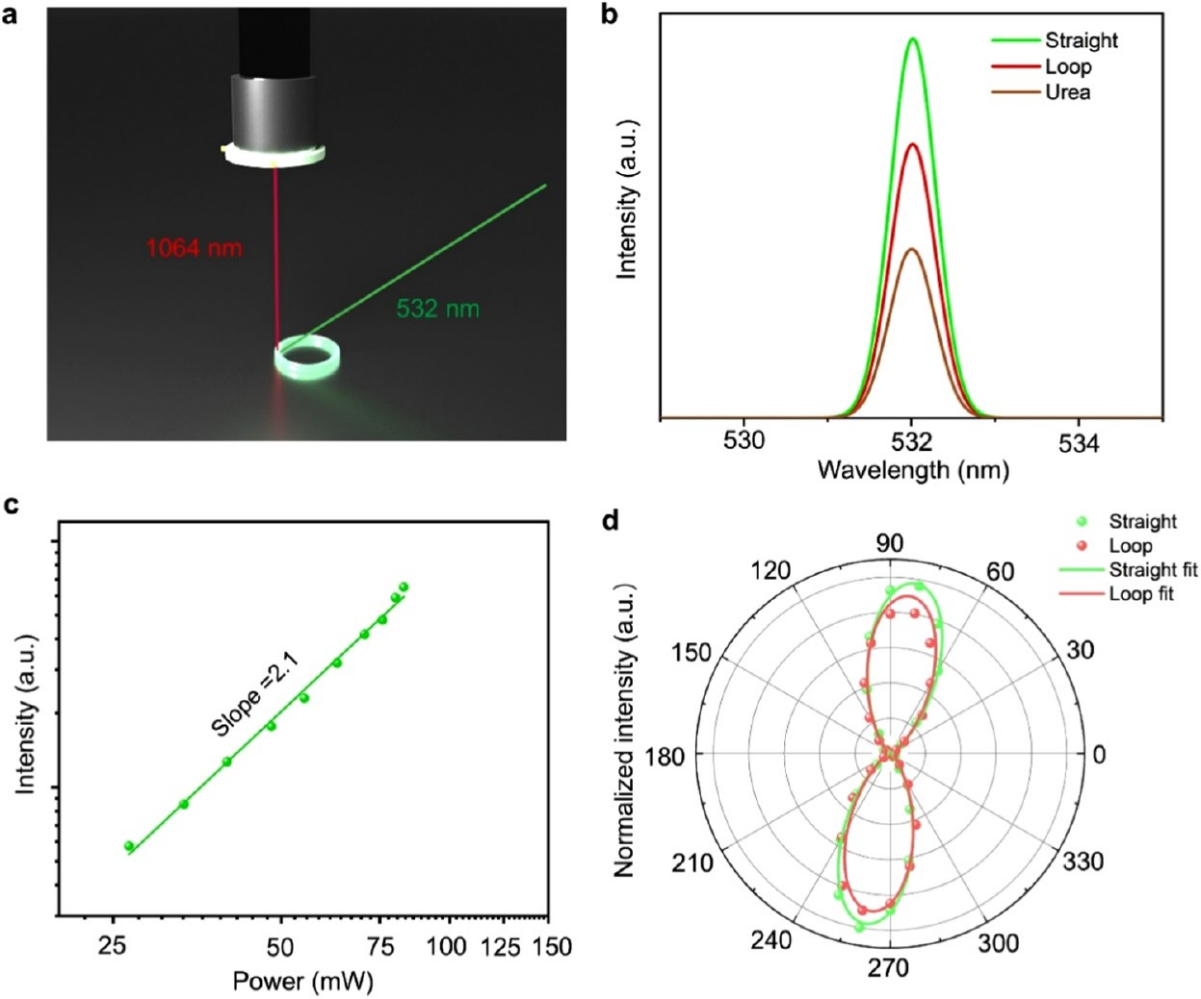
NLO properties of DPA. (a) Schematic diagram of the measurement of SHG properties of DPA crystal loops. (b) Comparison of the SHG intensities of a straight crystal of DPA and a DPA crystal loop with urea at 1064 nm under identical test conditions. (c) Logarithmic plot of the SHG intensity of a straight DPA crystal as a function of the incident power with pumping at 1064 nm. (d) Polarization dependence of the SHG intensity of a straight crystal and a crystal loop as a function of the linear polarization angle in parallel configuration.

Nonlinear optical (NLO) properties refer to the nonlinear interactions between light and matter, involving energy conversion of photons at different frequencies.^63−65^ The second harmonic generation (SHG) is one of the most important second-order NLO phenomena, where two incident photons combine to produce a single photon with double the frequency and thus double the energy.^65−67^ The crystal structure of DPA makes it particularly promising for SHG. When excited at 1064 nm, the SHG spectra of DPA crystals showed a strong response at 532 nm (Figures 7a and S34). The SHG intensity of a straight crystal was 2.03 ± 0.15 times stronger than that of the urea crystal under identical conditions (Figure 7b). The SHG signal of DPA had a quadratic relationship with the incident power, with a slope of about 2, indicating that the emergent light was generated by the SHG response of DPA (Figure 7c). Polarization-dependent SHG intensities at a parallel configuration showed that DPA crystals had a high anisotropy with the linear polarization pump and exhibited a two-leaf pattern with a maximum value at 82°/262°, approximately along the polar *c* axis (Figure 7d).^68,69^ The presence of both electron-donating (methyl) and electron-accepting (chloro) groups contributes to strong molecular polarization, with a calculated (DFT) permanent ground-state dipole moment of 5.51 D along the *a*/*c* axis. The molecules are assembled in an antiparallel arrangement along the *c* axis, resulting in cancellation of the dipole moment components along the *a* axis and constructive superposition along the *c* axis (Figure S35). This alignment generates a net dipole moment along the *c* axis.

We note that although the crystal loops exhibited highly efficient SHG, there was a slight decrease in SHG intensity compared with straight crystals. For instance, loops with an “equivalent strain” of 1% showed approximately 0.75 ± 0.12 times the SHG intensity of the straight crystals (Figure 7b). Moreover, the loops have an almost consistent polarization-dependent SHG intensity distribution with the straight crystals (Figure 7d). The SHG is related to the dipole moment, crystal symmetry, refractive index, etc.^67,70,71^ The bending may result in a slight change of crystal structure. Moreover, we noted that the crystallinity of the bent crystals decreased, which may affect the SHG intensity. The UV–vis absorption spectrum of solid DPA does not contain absorption >600 nm, and the crystals were photostable upon prolonged irradiation at 1064 nm although, as discussed above, they readily reacted when exposed to UV radiation (Figures 1c and S36). Given the strong SHG activity and prominent mechanical flexibility, DPA appears as a favorable mechanically compliant NLO material.

## Conclusions

The flexible fibrous material, DPA, reported here, combines abilities for bending during growth, mechanical bending, and photoinduced bending within a single molecular crystal. This material was found to crystallize as bent crystals or even crystals with completely circular habits of sizes that range from nanoscopic to macroscopic dimensions via template-free self-assembly from solution. Specifically, DPA grows as either single fibers or intergrowths of crystallites of different sizes from different solvents. The intergrowth of the constituent fibers may account for the bending of large crystals grown from a mixture of dichloromethane and ethanol. Furthermore, long, straight DPA crystals of centimeter size can be elastically bent along the (100) and (001) planes and can also be cut into fine fibers. The weak and dispersive vdW interactions provide the basis for structure adjustment during crystal growth and mechanical bending. The NLO properties of both straight and annular crystals were also studied. Compared to the straight crystals, the crystal loops with “equivalent strain” of about 1% exhibited a slight decrease of SHG intensity, which was still up to 1.52 ± 0.12 times stronger than that of urea. We believe that this work can guide the preparation of crystal loops of other materials and particularly, flexible crystalline NLO materials.

## Experimental Section

### Materials

All chemicals for the synthesis and crystallization were purchased from commercial sources and used without further purification. The purity of 2,3-dichloroisonicotinaldehyde and 2-(3,5-dimethylphenyl)acetonitrile used for synthesis was ≥98%. Dichloromethane, ethyl alcohol, ethyl acetate, and toluene used for crystallization were of purity ≥99.5%, 99.7%, 99.9%, and 99.5%, respectively.

### Preparation of DPA

2-(3,5-Dimethylphenyl)acetonitrile (0.2904 g, 2 mmol) and 2,3-dichloroisonicotinaldehyde (0.3520 g, 2 mmol) were added to 10 mL of ethanol, and 5 mg of NaOH was added to the solution. Cyan-colored powder precipitated by stirring for 1.5 h at 60 °C, and then the mixture was kept at 5 °C to improve the yield. The product was collected by filtration and washed with ethanol to obtain a cyan-colored solid (0.359 g, 59.2%).

### Preparation of DDC

DPA was ground into a microcrystalline powder and spread out as a very thin layer. The material was irradiated with a 365 nm OLED UV lamp (10 W) for 2 h. The irradiated sample was recrystallized from a mixture of ethanol and dichloromethane.

### Crystal Growth

Recrystallization of DPA was performed from different solvents and using different crystallization methods. The bent crystals of micro- and nanosize were obtained from toluene or ethyl acetate. From toluene, bent crystals were prepared by slow evaporation of a solution with a concentration of 1 mg mL^–1^ at 25 °C. From ethyl acetate, bent crystals were obtained by slow evaporation of solutions with concentrations of 0.5, 1, 1.5, 2, and 2.5 mg mL^–1^ at 25 °C. Crystals of DPA of large size were obtained from a mixture of dichloromethane and ethanol (1:0.75) with concentrations from 1.5 to 4 mg mL^–1^ at 25 °C. Crystals were also obtained by layering ethanol (6 mL) on top of 1 mL of dichloromethane solution of DPA with concentrations of 10–20 mg mL^–1^ at 5 °C. The crystals were usually obtained within 2 weeks.

### UV-Induced Photomechanical Effects of DPA

For observation of the photomechanical response, the DPA crystals were placed on a plastic base and irradiated with a 365 nm OLED ultraviolet lamp. To study the photochemical response kinetics and reversibility, the crystal was glued onto a metal needle and irradiated on the (100) facet with 365 nm UV light at an intensity of approximately 18 mW cm^–2^.

## Supporting Information Available

Additional methods, figures, tables, and X-ray crystallographic data (PDF)
Movie S1: Growth-induced bending in ethyl acetate (MP4)
Movie S2: Growth-induced bending in ethyl acetate (MP4)
Movie S3: Growth-induced bending in a mixture of ethanol and dichloromethane (MP4)
Movie S4: Growth-induced bending in a mixture of ethanol and dichloromethane (MP4)
Movie S5: Mechanical bending on the (100) and (001) planes (MP4)
