# Supplementary material for: Flexible Organic Crystalline Fibers and Loops with Strong Second Harmonic Generation
Source: J Am Chem Soc. 2025 Mar 20;147(13):11346–58. doi: 10.1021/jacs.5c00598 (PMC11969535; doi:10.1021/jacs.5c00598)
Supplement: Supplementary file 1 — ja5c00598_si_001.pdf [file ja5c00598_si_001.pdf]

Supporting Information

Flexible Organic Crystalline Fibers and Loops with Strong Second Harmonic Generation

Jiawei Lin,[1#] Shi Tang,[1#], Liang Li,[3,4] Liwen Fang,[5] Qun Zeng,[5] Guangxu Sun,[5] Songgu Wu,[1,2*]
Panče Naumov,[3,6,7,8*], Junbo Gong[1,2*]

[1]*School of Chemical Engineering and Technology, State Key Laboratory of Chemical Engineering, Tianjin University, Tianjin 300072, China*

[2]*Haihe Laboratory of Sustainable Chemical Transformations, Tianjin 300192, China*

[3]*Smart Materials Lab, New York University Abu Dhabi, PO Box 129188, Abu Dhabi, UAE*

[4]*Novel Materials Development Lab, Sorbonne University Abu Dhabi, PO Box 38044, Abu Dhabi, UAE*

[5]*XtalPi Inc., Shenzhen Jingtai Technology Co., Ltd., Shenzhen 518100, China*

[6]*Center for Smart Engineering Materials, New York University Abu Dhabi, PO Box 129188, Abu Dhabi, UAE*

[7]*Research Center for Environment and Materials, Macedonian Academy of Sciences and Arts, Bul. Krste Misirkov 2, MK–1000 Skopje, Macedonia*

[8]*Molecular Design Institute, Department of Chemistry, New York University, 100 Washington Square East, New York, NY 10003, USA*

[#]These authors contribute equally to this work.

Supplementary Methods

1. Single-crystal X-ray diffraction analysis

Diffraction data were collected on a Rigaku XtaLAB FR-X system with VariMax optic coupled with the FR-X rotating anode generator using Cu (λ = 1.54184 Å) or Mo (λ = 0.71073 Å) radiation, equipped with an Oxford Cryosystems 700 series cold N_2 gas stream cooling system. Data integration and reduction were undertaken with CrysAlisPro.[1] The intensities were corrected for Lorentz and polarization effects, and an empirical absorption correction was applied. The structures were solved by intrinsic phasing method using SHELXT[2] then refined and extended with SHELXL[3] in Olex2.[4] Non-hydrogen atoms were refined anisotropically. Carbon-bound hydrogen atoms were included in idealized positions and refined using a riding model.

2. Nanoindentation test

The nanoindentation measurements on DPA crystals were performed by using Agilent G200 nanoindenter equipped with an XP head and a Berkovich diamond indenter. The indenter tip had a nominal radius of about 20 nm with the pyramidal faces forming an angle of 65.3° with the vertical axis. Single crystals were glued onto a microscope slide using a drop of cyanoacrylate adhesive prior to the experiments. The (100) plane was indented to a peak load of 0.5 mN in all tests, and the *P–h* curves were analyzed by using the standard Oliver−Pharr method[5,6] to extract the hardness and Young's modulus. The Poisson's ratio was assumed to be 0.18.

3. Scanning electron microscopy (SEM)

Field-emission scanning electron microscopy was performed by using Apreo S LoVac scanning electron microscope (Thermo Fisher Scientific) to inspect the small crystals morphology grown from ethyl acetate and toluene. The samples grown on silicon were coated with gold to enhance the conductivity. The operating voltage was 2−5 kV. The large crystals grown from dichloromethane/ethanol mixture were inspected by using a TM3000 scanning electron microscope (Hitachi) with an accelerating voltage of 15 kV.

4. Micro-Raman spectroscopy

Raman spectroscopy was used to distinguish the vibrational signature of the straight and bent crystals. The spectra were recorded using a Raman microscope DXR (Thermo Fisher Scientific), equipped with a 532 nm excitation laser operating at 2 mW and with a 50 µm slit.

5. Fluorescence spectroscopy

A steady-state/transient fluorescence spectrometer (FLS1000, Edinburgh, UK) was used to record the fluorescence spectral data for single crystals DPA and DDC. For each group of samples, the single crystals with good quality were selected. The samples were flatly placed on the quartz sample tank to cover the surface, covered with quartz sheets, and fixed with clips.

6. UV−vis absorption spectroscopy

The UV−vis absorption was measured by LAMBDA 750 (Perkin Elmer).

7. Hirshfeld surface and energy framework analysis

Hirshfeld surface 2D fingerprint plots and energy framework analysis were plotted using the CrystalExplorer software (ver. 17.5).[7,8] The Energy Frame was calculated at B3LYP/6-31G(d,p) level of dispersion-corrected density functional theory basis set. The energy components calculated within this method are electrostatic, polarization, dispersion, and exchange-repulsion and finally the total interaction energy. It was calculated by adding corresponding interaction energies between a given molecule in one layer and all interacting molecules in a neighboring layer or within the same layer within 3.8 Å. The tube size used in the energy framework was 100 and lower energy threshold (cut-off) value was set to -5 kJ/mol.

8. Calculations

Interaction region indicator (IRI) analysis

IRI is essentially the gradient norm of electron density weighted by scaled electron density and can be defined as[9]

$$\mathrm{IRI}\,(\mathrm{r}) = \frac{|\nabla\rho(r)|}{[\rho(r)]^{a}}$$

Where ρ is electron density and r is coordinate vector, a is an adjustable parameter, $a = 1.1$ is adopted for standard definition of IRI, ∇ is the gradient operator, $|\nabla\rho(r)|$ is the norm of electron density gradient, and $sign(\lambda_2)$ is the sign of second Hessian eigenvalue times the electron density.

The region with a relatively high ρ and thus a large magnitude of $sign(\lambda_2)\,\rho$ indicates a relatively strong interaction. On the contrary, there are no noticeable interactions when the area with a low ρ and thus a small $sign(\lambda_2)\,\rho$. The interactions can be at most attributed to the very weak interatomic vdW interactions.[10]

The $sign(\lambda_2)\rho$ function is projected on the IRI iso-surface with different colors to distinguish the action intensity and characteristics of different regions. The green represents van der Waals interactions, red indicates repulsive/steric interactions and blue indicates the robust, attractive interactions such as hydrogen bonding and halogen bonding.

The theoretical calculation of weak interactions was calculated with the accuracy of B3LYP/6-311+G(d,p) using Gaussian 09 software. IRI analysis was visualized using Multiwfn 3.7[11] and VMD programs[12], and plotted using gnuplot[13].

Calculation of the molecular dipole moment

The ground state molecular dipole moment was calculated based on B3LYP/6-31G+(d,p) using Gaussian 16[14] and visualized by GaussView[15].

Calculation of the strain-energy and surface energy

The equilibrium structure was calculated for DPA periodic dispersion-corrected density functional theory method as implemented in VASP[16]. It uses the projector-augmented wave method with plane-wave basis sets and PAW pseudo potentials. The PBE and generalized gradient approximation (GGA) was used for the exchange-correlational functional coupled with the D3 dispersion-correction. In all calculations, the cut-off energy for the planewave basis set was set at 520 eV. The convergence threshold for energy was set at 0.001eV and for force was 0.001ev/A. The two-stage geometry optimization was performed to calculate the equilibrium structure in different strains. Firstly, the resolved structure was optimized. Then, it was followed by the optimization of atomic positions with the lattice parameters fixed after application of the tensile strain from -5% to 5%. For the calculation of the surface energy, firstly, the cell structure was optimized. Then, the structure was further optimized with surface molecules on (001) or (00-1) plane relaxed and other molecules were fixed. It was followed by the solvation. The single point energy of solvation was calculated based on VASPSOL. The used dielectric constant of toluene and ethyl acetate for calculation was 2.38 and 6.02, respectively.

Calculations of the solvation free energy

The calculation of solvation free energy was calculated based on Materials Studio 2019. Firstly, the DPA and solvent molecules were optimized to minimize energy. The amorphous cell was built with 300 solvents and a DPA molecules. For the system of mixed solvents, the ratio of molecules of dichloromethane to ethanol is 1:1. An atom-based simulation method was employed to calculate the van der Walls and electrostatic interactions with a cutoff distance of 15.5 Å. Geometry optimization with >5000 iterations was carried out to minimize energy of the amorphous cell. Next, molecular dynamics were performed with NVT ensemble (fixed number of particles (N), volume(V), and temperature (T)) and NPT ensemble (constant particle numbers (N), pressure (P), and temperature (T)), respectively at 298.15 K and 101.3 kPa. The calculated time of both NVT and NPT ensemble was 1 ns. The last frame of NPT was used to calculate the free energy of solvation.

9. Transmission electron microscopy (TEM)

Tansmission electron microscopy (TEM) and high-resolution TEM (HRTEM) imaging were carried out using Tecnai G2 F20 at 200 kV (Philips Netherlands Limited). High angle annular scanning transmission electron microscopy (HAADF-STEM) imaging was carried out on Thermofisher Talos F200X at 200 kV.

10. Observation of the photomechanical effects

For observation of the photomechanical effects, DPA crystals were placed on a black plastic base, and irradiated with an OLED ultraviolet lamp (365 nm). Their dynamic behaviors were observed by using optical microscope (Nikon, SMZ745T).

11. Measurement of SHG

SHG characterization was measured by the WITec alpha300 Raman system using 1064 nm excitation (15 ps, 80 MHz). For angle-resolved measurement, a laser was rotated by a motorized stage (step of 8°).

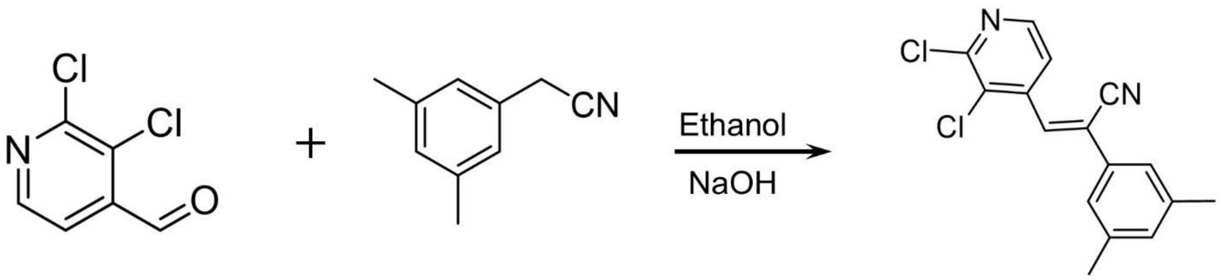

Scheme S1. Chemical synthesis of DPA.

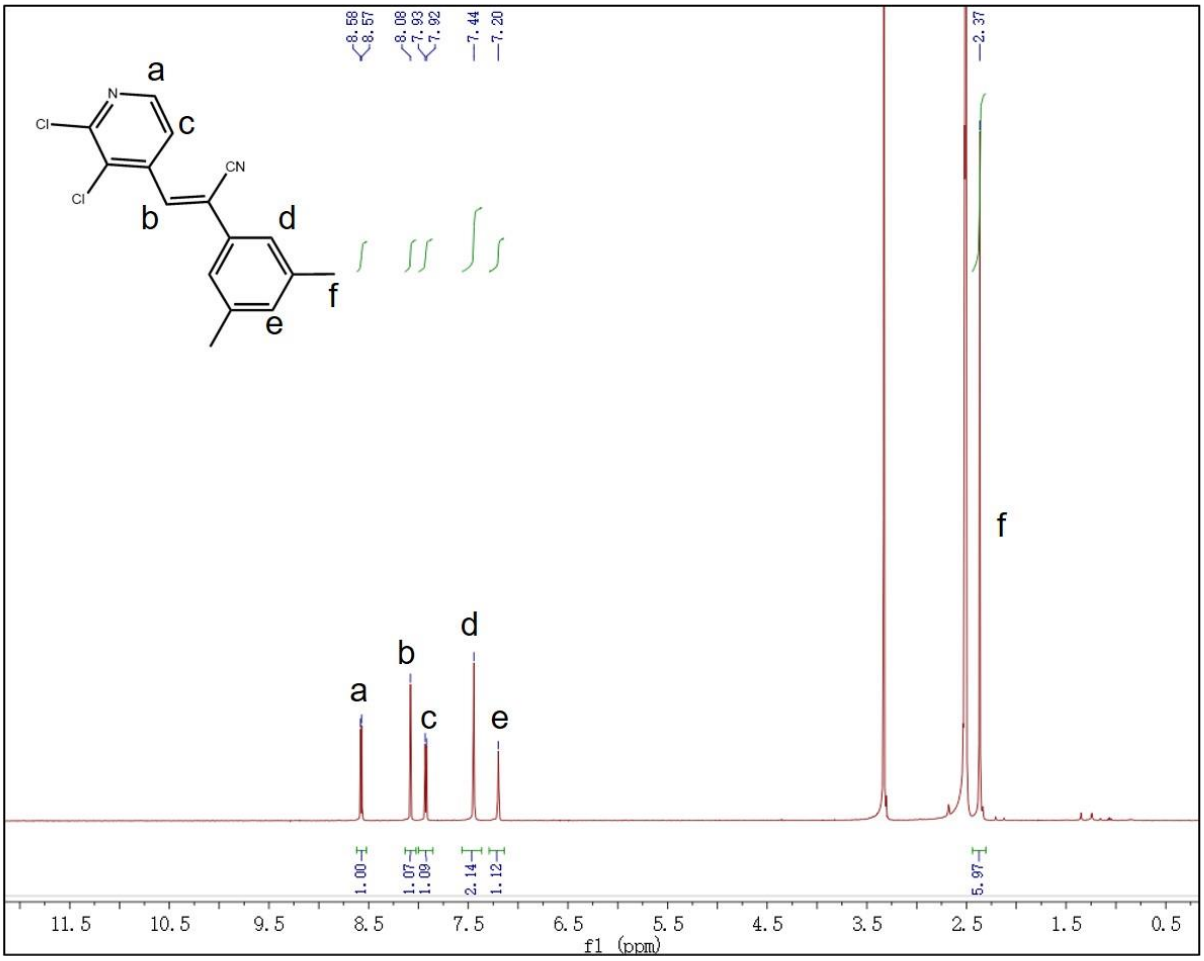

Figure S1. ^{1}H NMR spectrum of DPA in DMSO-d_6 (400 MHz). ^{1}H NMR (400 MHz, DMSO-d_6) δ/ppm = 8.57 (d, J = 5.0 Hz, 1H), 8.08 (s, 1H), 7.93 (d, J = 5.4 Hz, 1H), 7.44 (s, 2H), 7.20 (s, 1H), 2.37 (s, 6H).

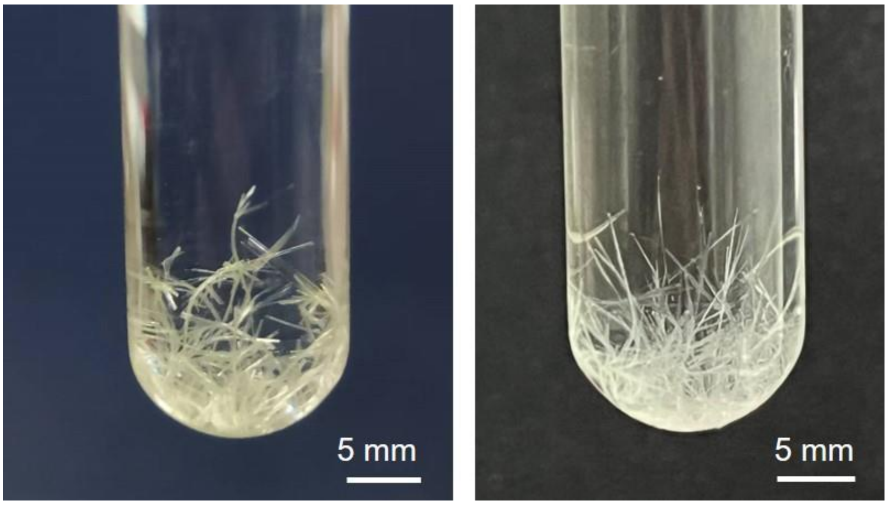

Figure S2. Optical photographs of DPA crystals obtained by layering ethanol on the top of a dichloromethane solution of DPA.

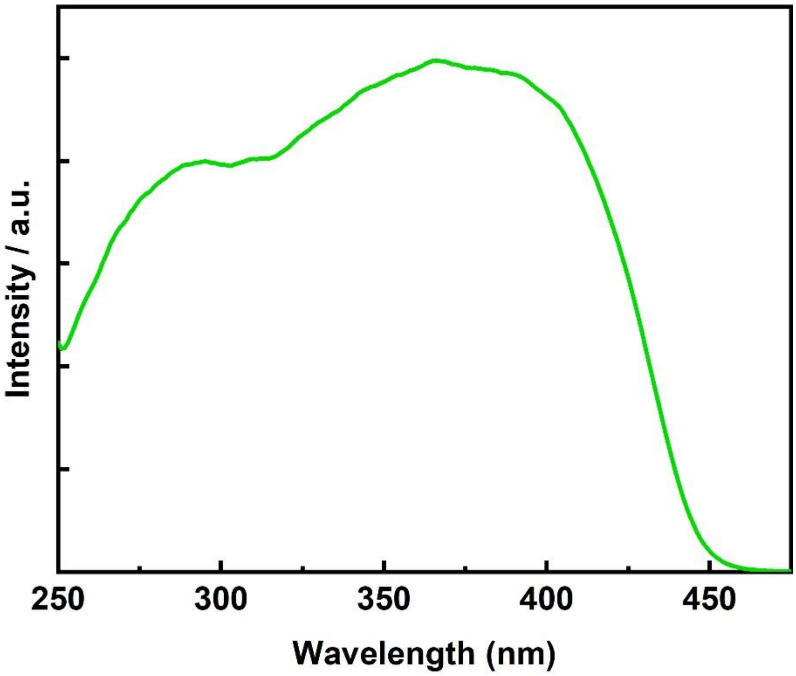

Figure S3. Excitation spectrum of solid DPA at room temperature.

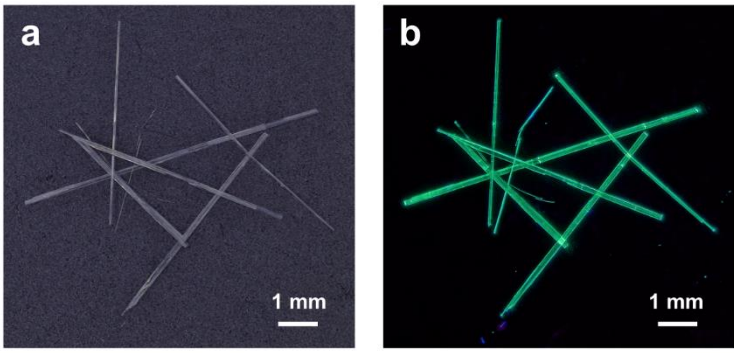

Figure S4. Optical photographs of the needle-like single crystals of DPA. (a) DPA crystals taken under room light. (b) DPA crystals under a 365 nm UV light.

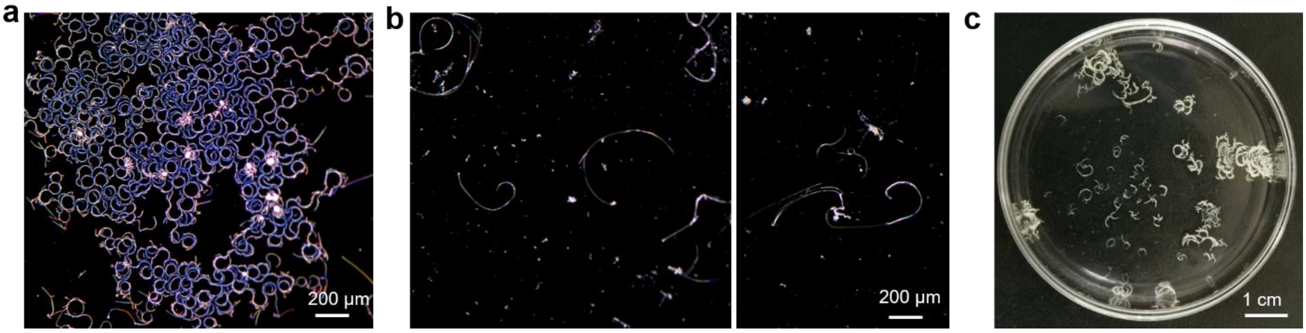

Figure S5. Optical photographs of DPA crystals obtained through solvent evaporation. (a) Bent crystals from ethyl acetate. (b) Bent crystals from toluene. (c) Bent crystals from the mixture of dichloromethane and ethanol.

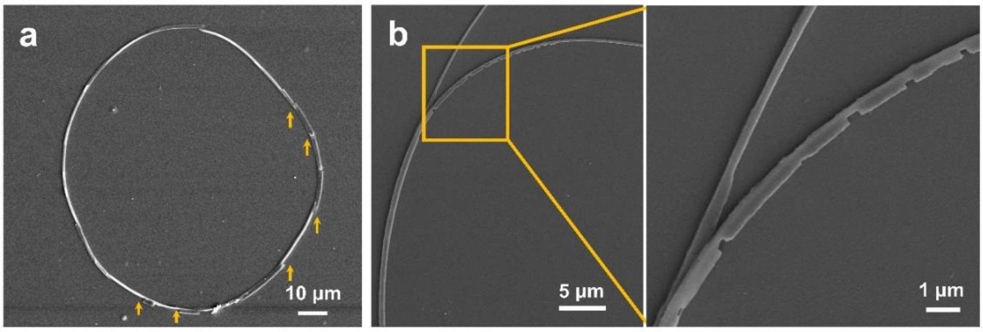

Figure S6. SEM images of the surface morphology of crystals of DPA obtained from ethyl acetate.

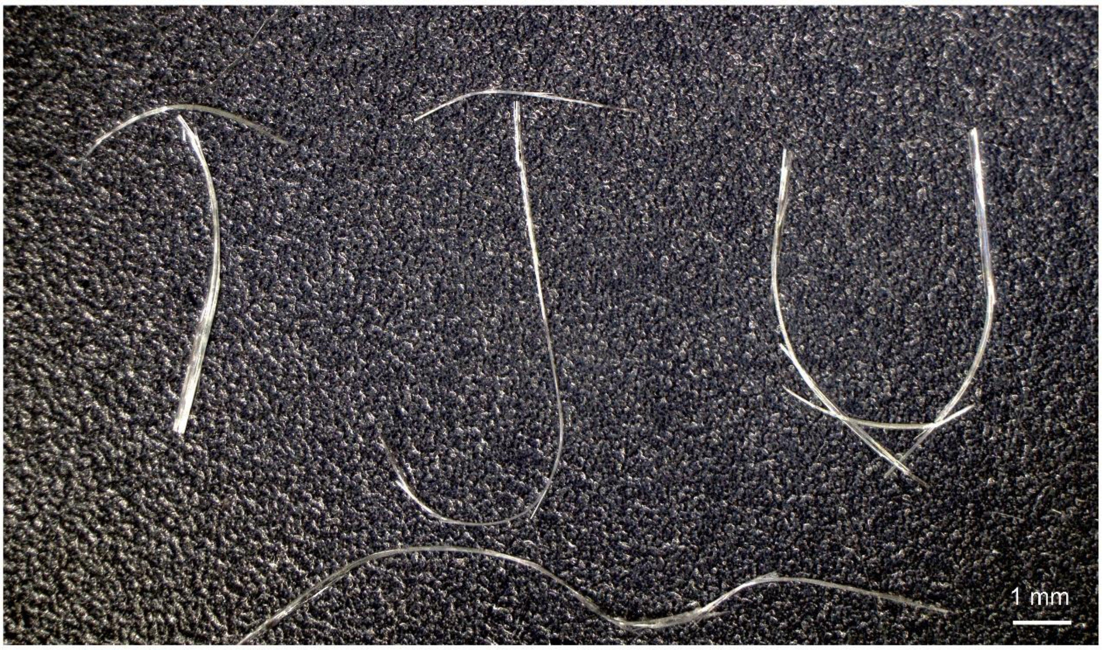

Figure S7. Optical micrographs of DPA crystals shaped as letters "TJU" showing the flexibility of the crystals.

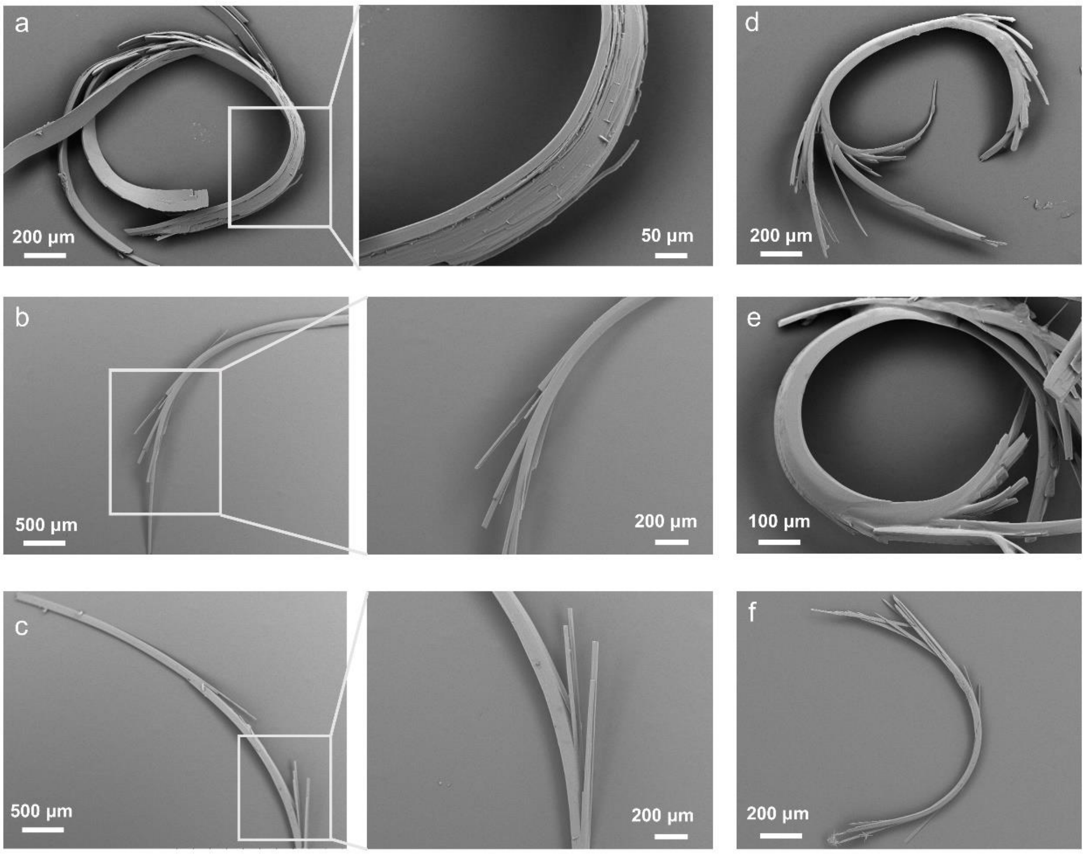

Figure S8. SEM images of bent crystals grown from a mixture of ethanol and dichloromethane.

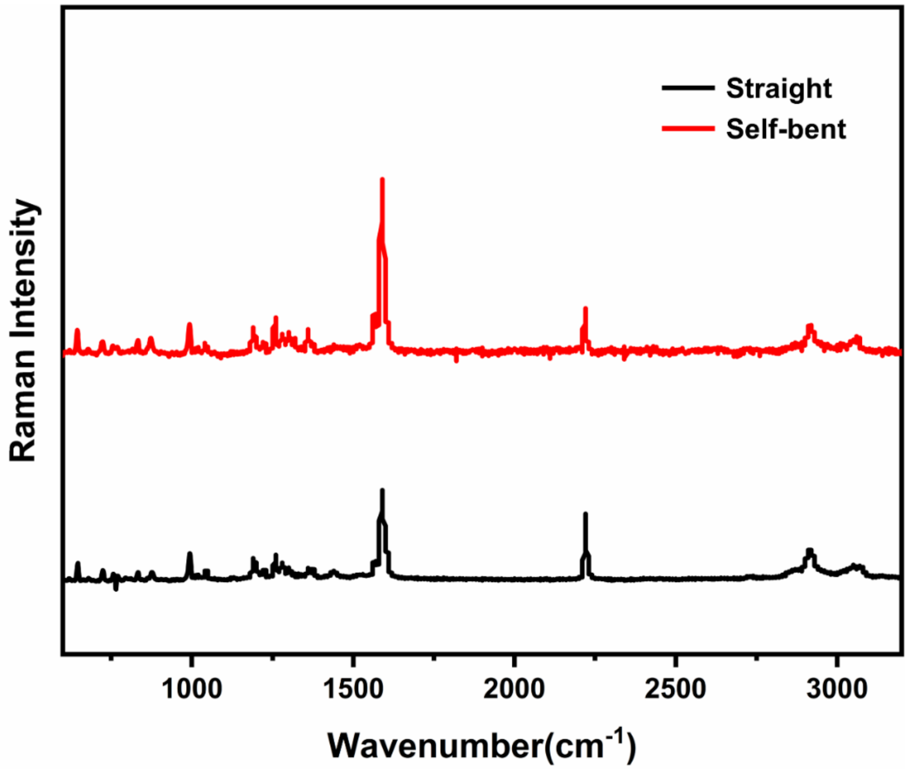

Figure S9. Micro-Raman spectra of the straight and as-grown bent crystals.

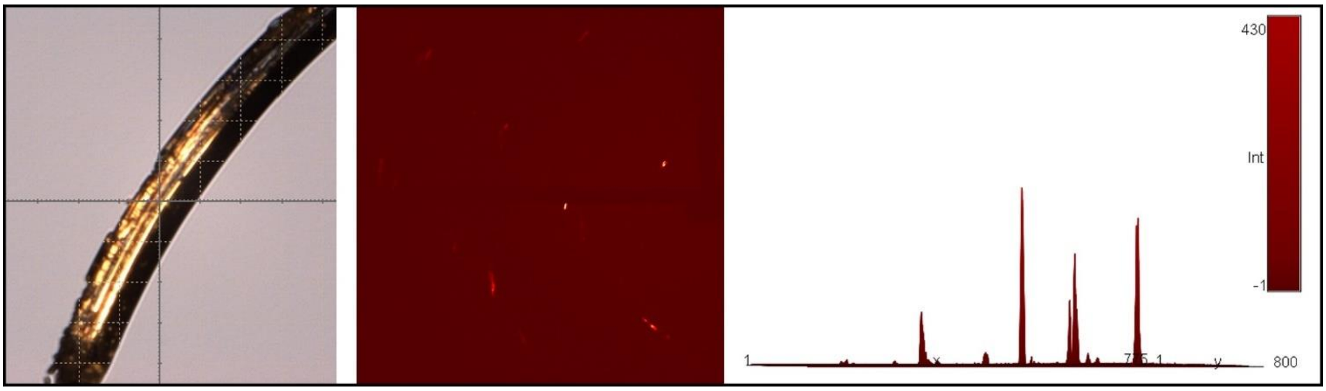

Figure S10. Optical images and exemplary Bragg diffraction images of as-grown bent crystals.

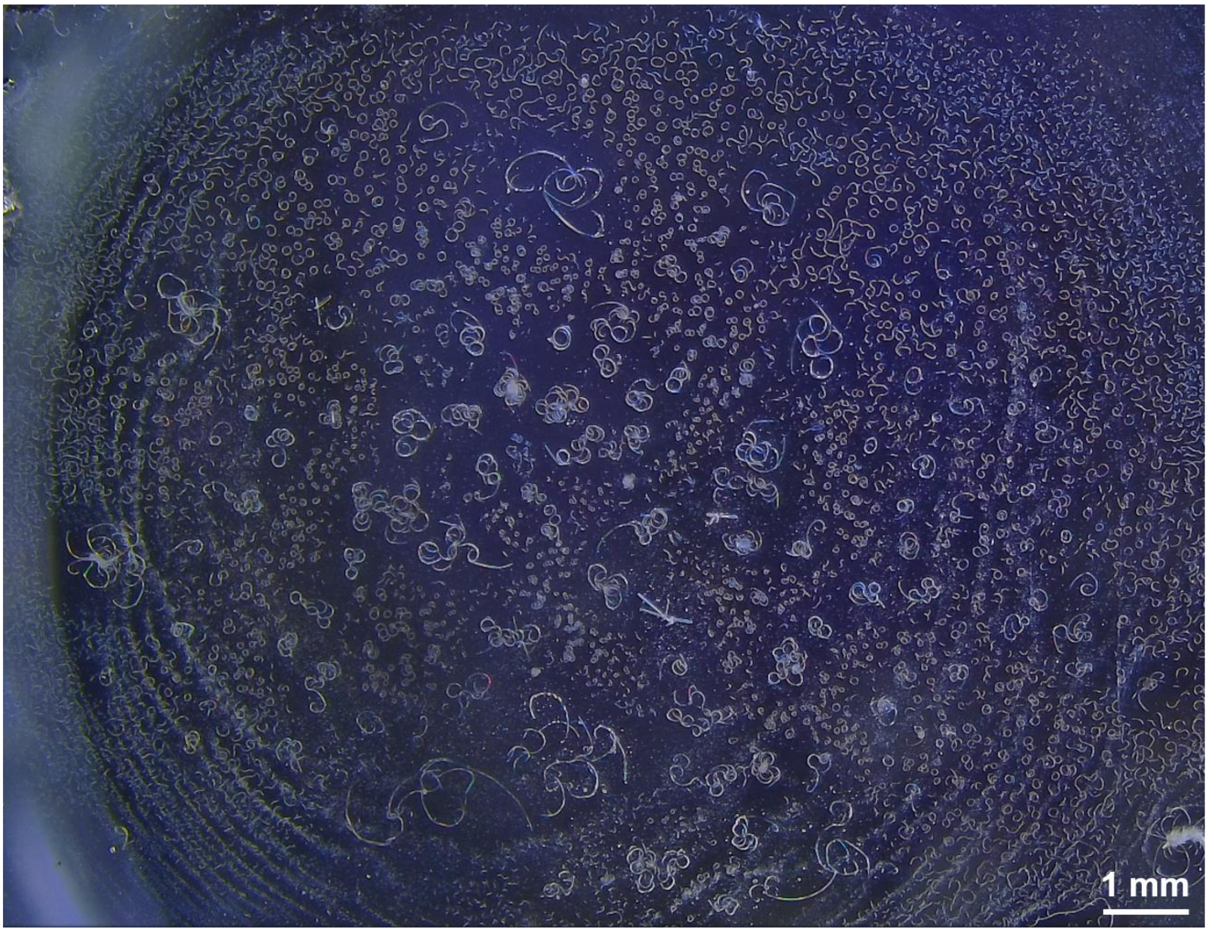

Figure S11. Optical micrographs of DPA crystals obtained from ethyl acetate.

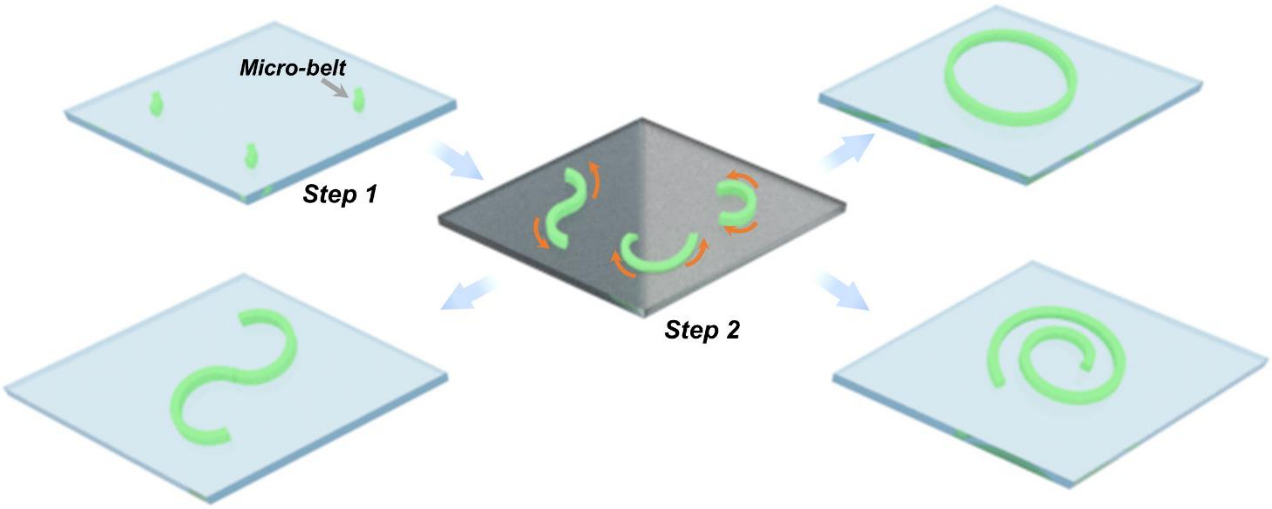

Figure S12. Schematic diagram of the morphology evolution of three typical bent crystals. Step 1: DPA crystals derivate into micro-belt. Step 2: The micro-belt grows with a certain curvature at both ends

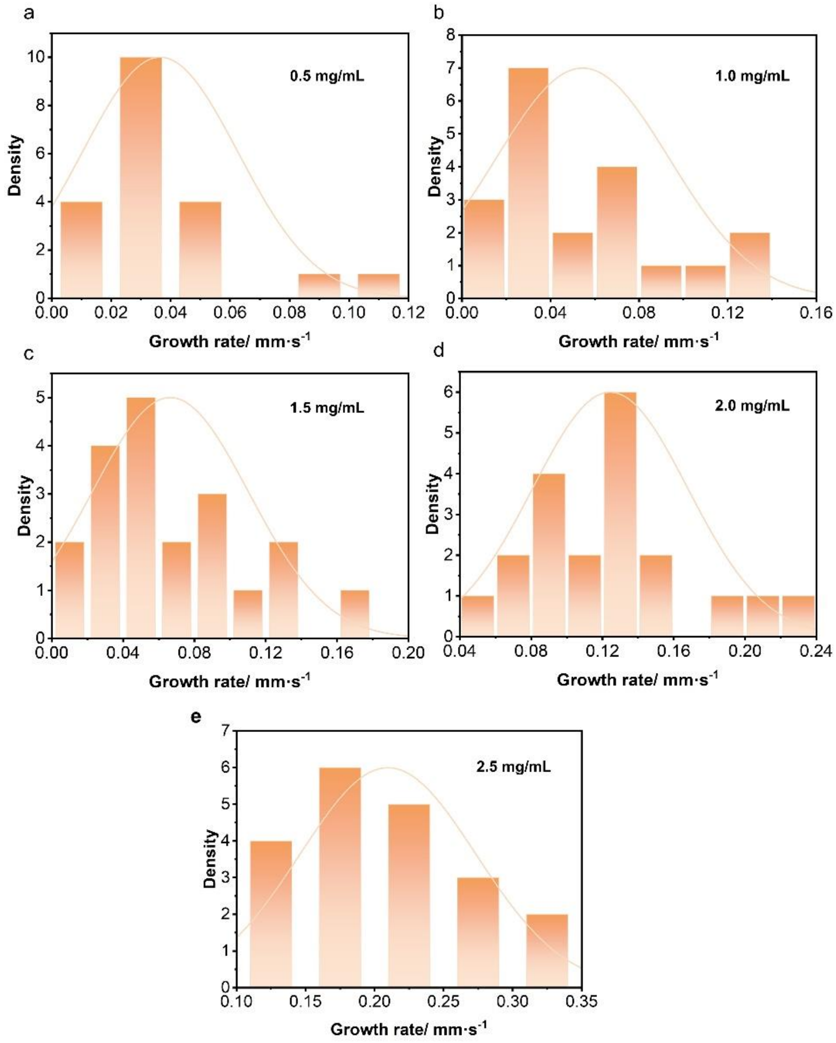

Figure S13. Distribution of the growth rate across different crystallization concentrations in ethyl acetate. (a) 0.5 mg/mL, (b) 1.0 mg/mL, (c) 1.5 mg/mL, (d) 2.0 mg/mL, (e) 2.5 mg/mL.

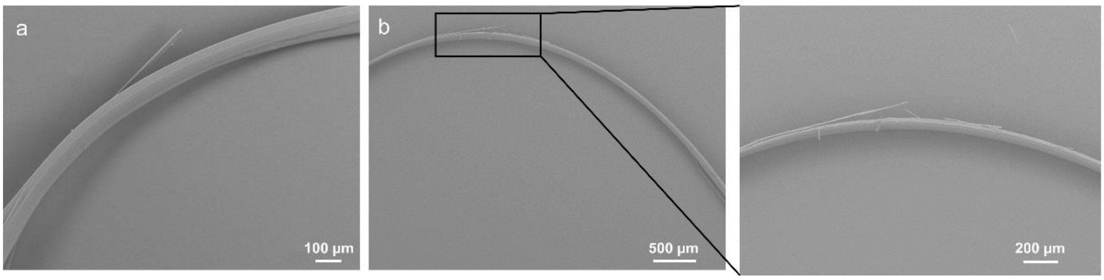

Figure S14. SEM image of a bent crystals. (a) Bending on the (001) plane. (b) Bending on the (100) plane.

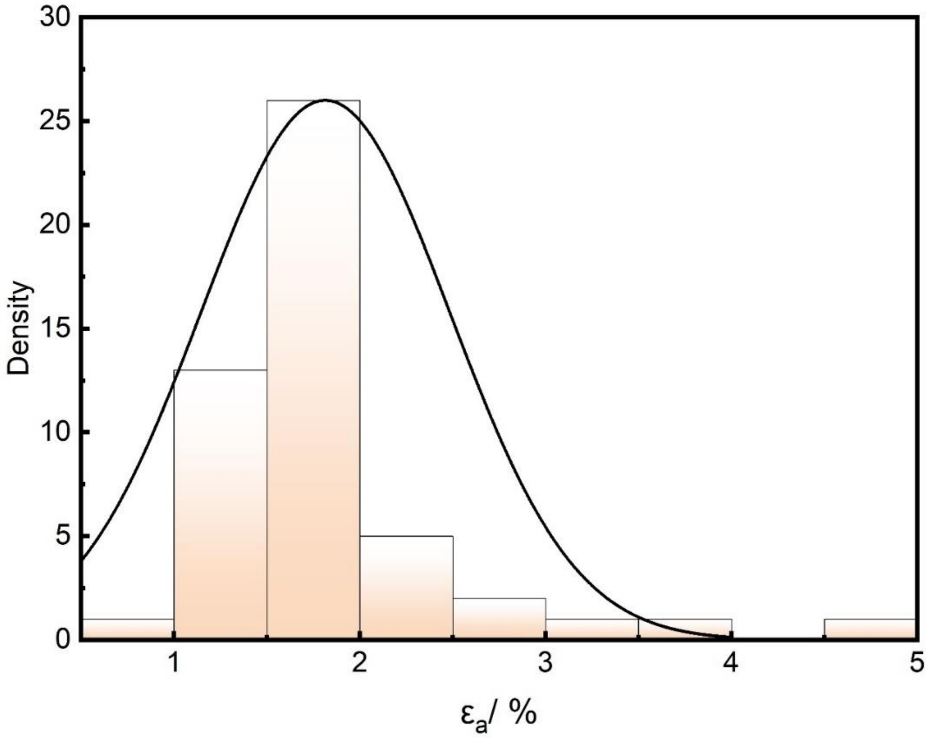

Figure S15. Distribution of Elastic strain limit of the DPA crystals.

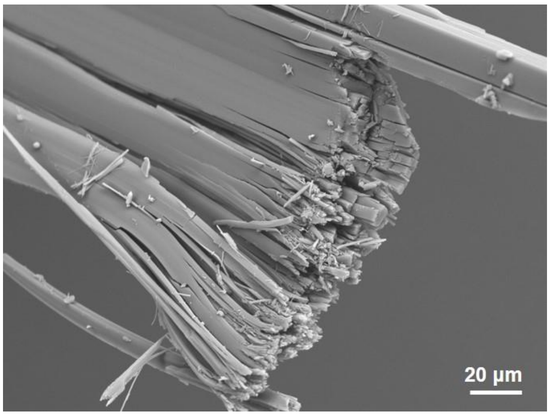

Figure S16. SEM image of the fracture surface.

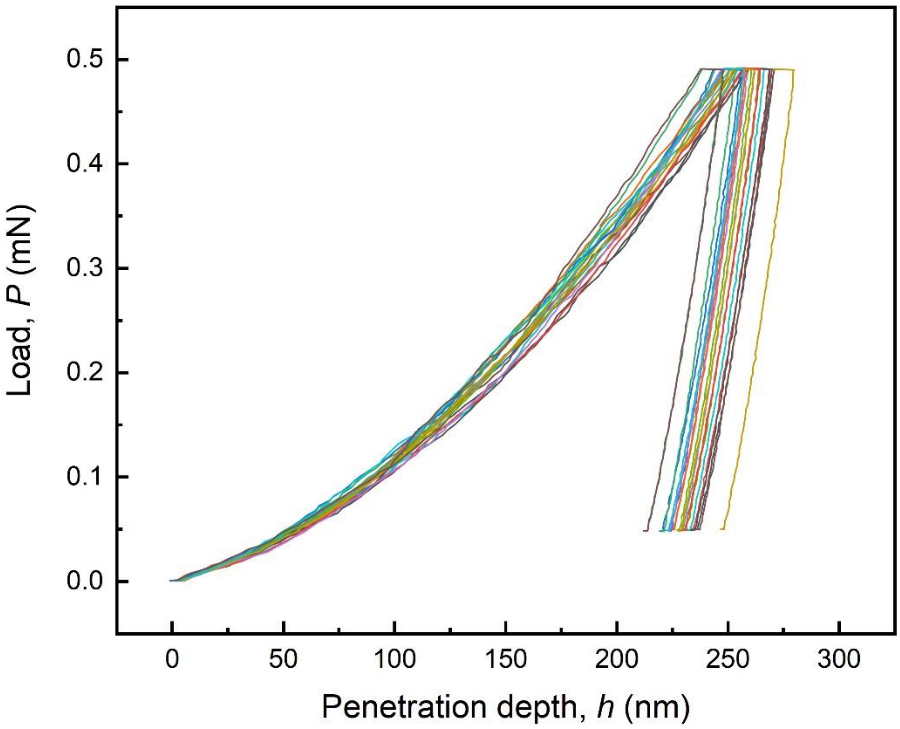

Figure S17. Representative load-depth (*P*–*h*) curves obtained using a 0.5 mN load on the (100) plane.

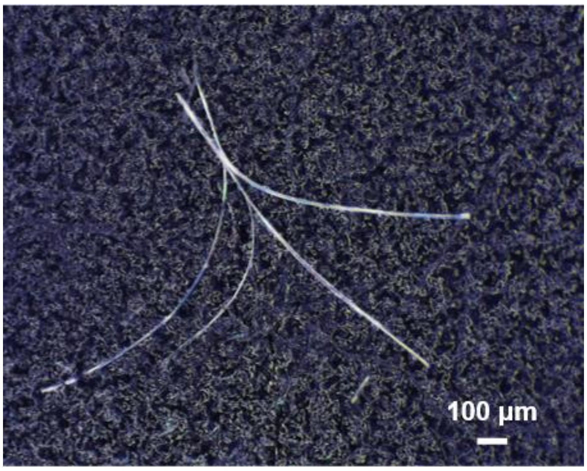

Figure S18. Images of the elastic deformation of splitting crystals.

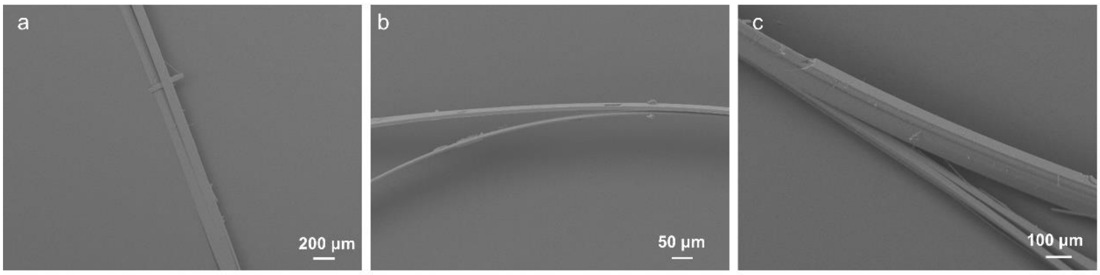

Figure S19. SEM images of mechanical splitting crystals.

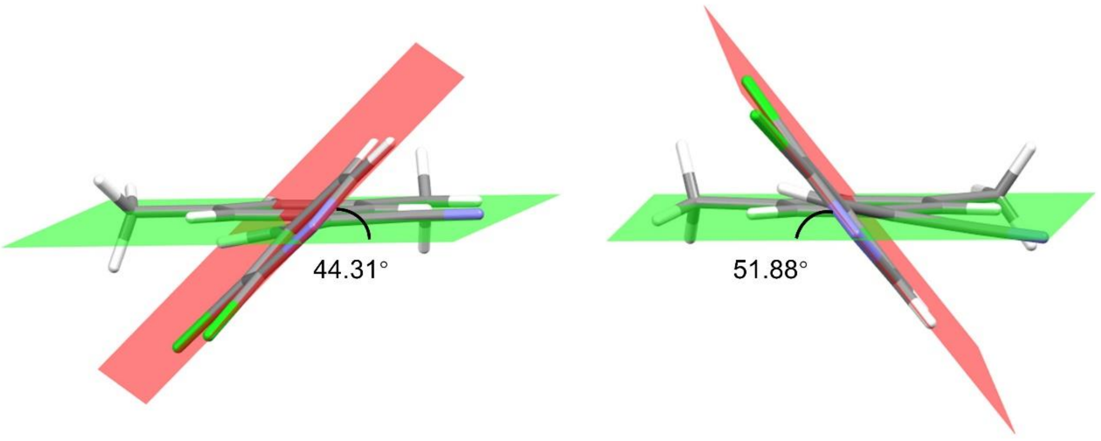

Figure S20. The dihedral angle between the benzene ring and pyridine ring.

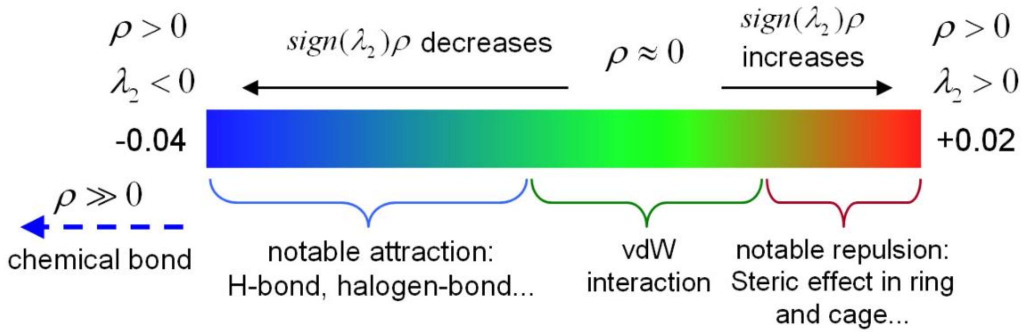

Figure S21. Standard coloring method and chemical explanation of *sign(*λ_2*)* ρ on IRI isosurfaces.[8]

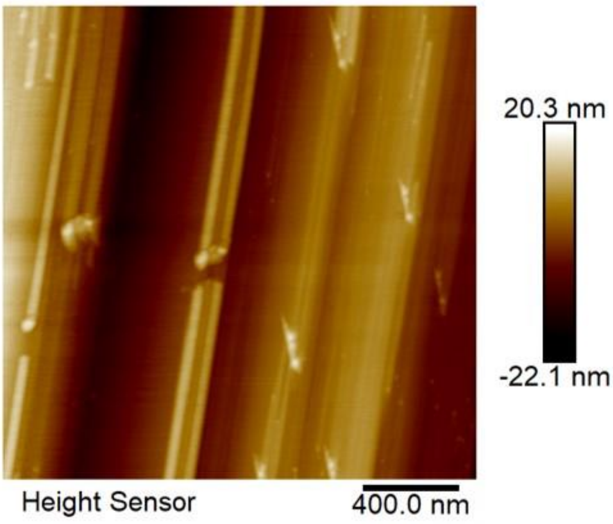

Figure S22. Surface topography of the straight crystals observed by atomic force microscopy.

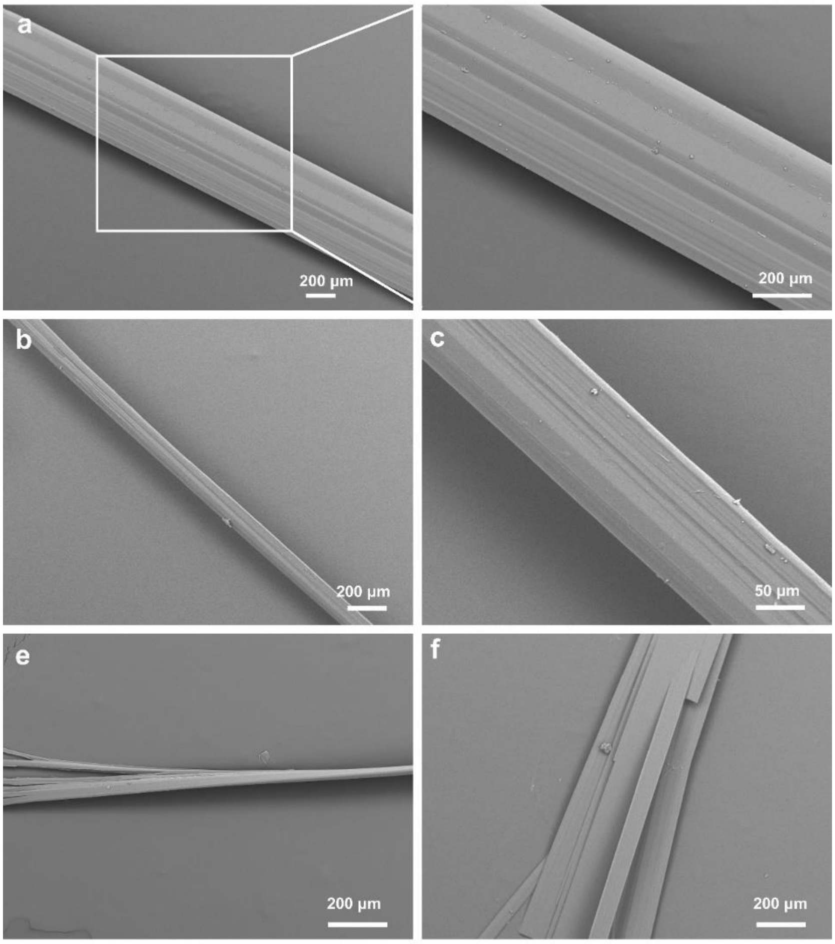

Figure S23. SEM images of crystals with straight habit.

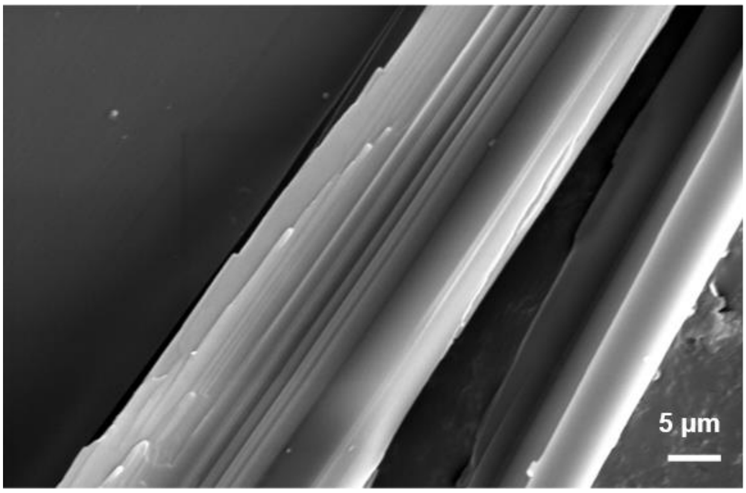

Figure S24. SEM image of split crystalline fibers.

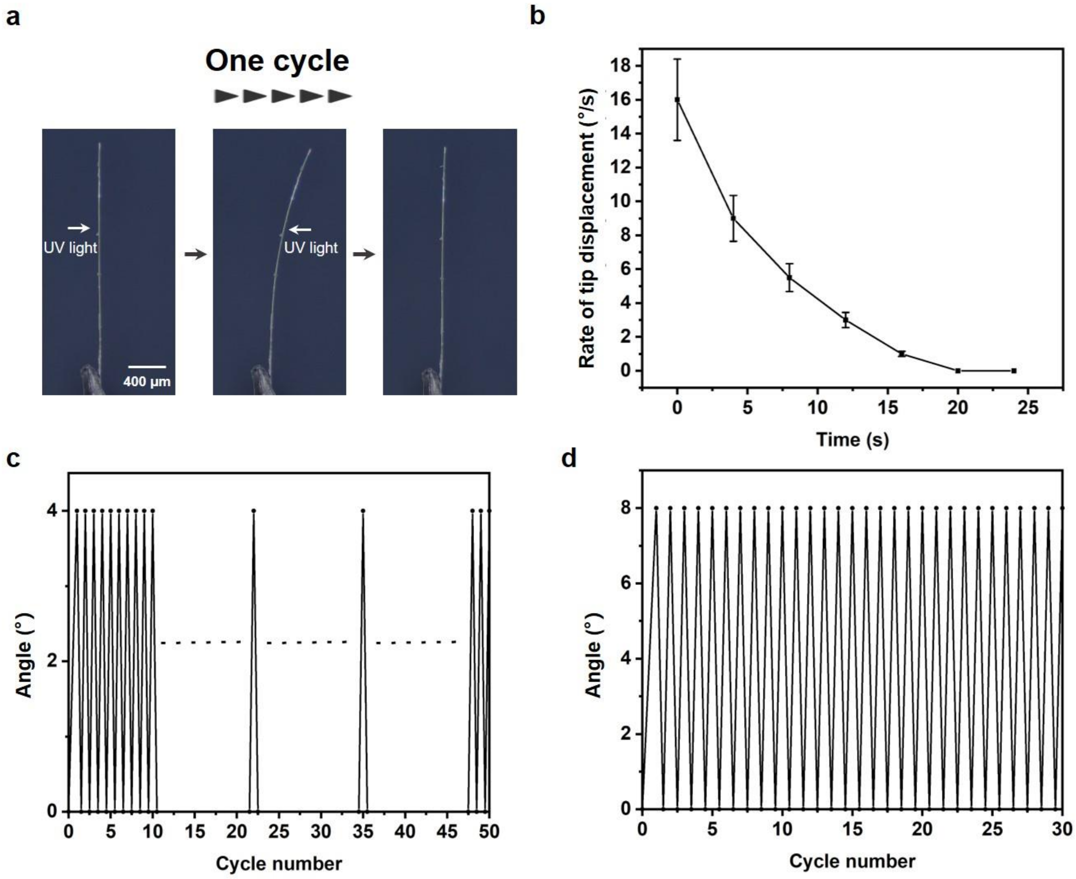

Figure S25. Dynamics and reversibility of photomechanical bending of DPA crystals. (a) Reversible bending of a needle-like DPA crystal upon alternative irradiation with a 365 nm light from opposite sides. (b) The rate of bending, illustrated by the change in deflection angle with time. (c, d) Reversibility of the macroscopic deformation recorded at different displacement angles: (c) 4°, (d) 8°.

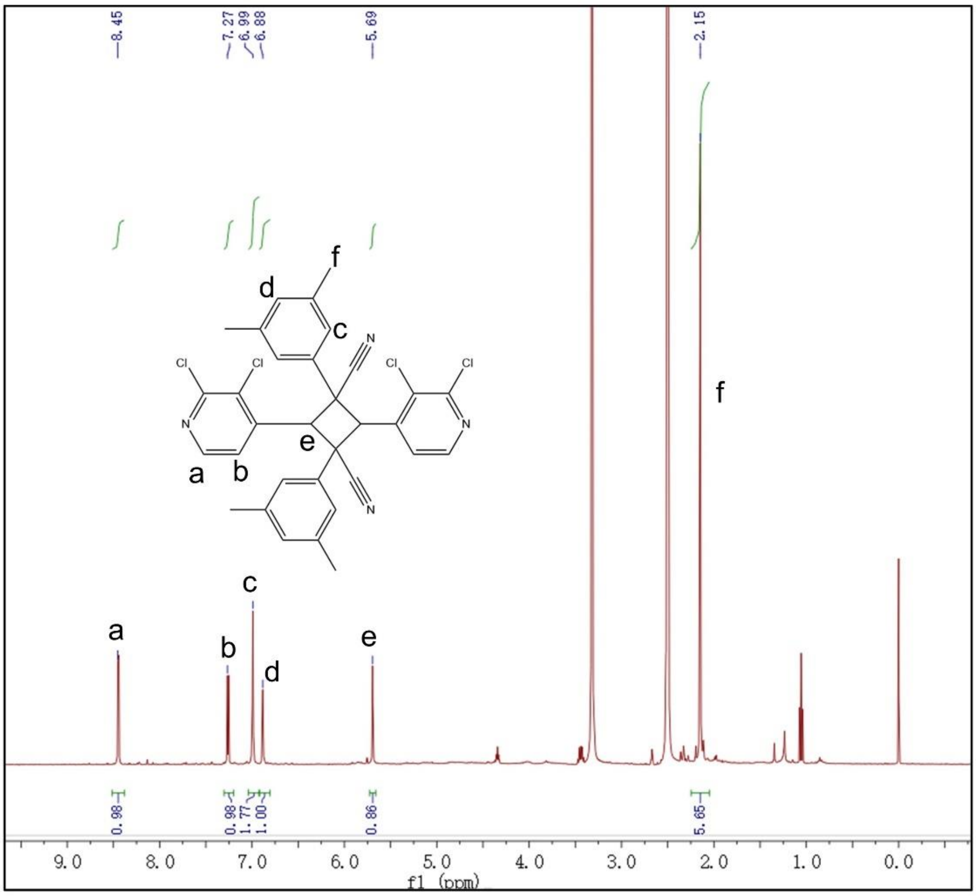

Figure S26. ^{1}H NMR spectrum of DDC in DMSO-d_6 (400 MHz). ^{1}H NMR (400 MHz, DMSO-d_6) δ/ppm = 8.45 (d, J = 5.1 Hz, 1H), 7.27 (d, J = 5.1 Hz, 1H), 6.99 (s, 2H), 6.88 (s, J = 20.9 Hz, 1H), 5.69 (s, 1H), 2.15 (s, 6H).

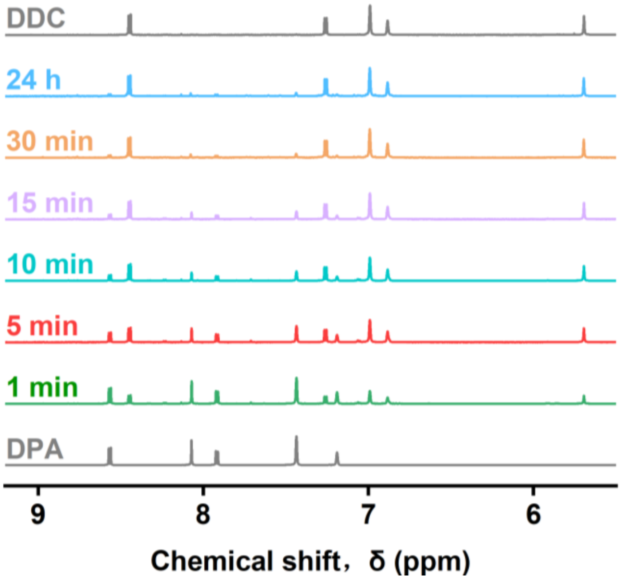

Figure S27. The photocycloaddition reaction of DPA monitored by ^{1}H NMR spectroscopy and spectral comparison with the dimer, DDC.

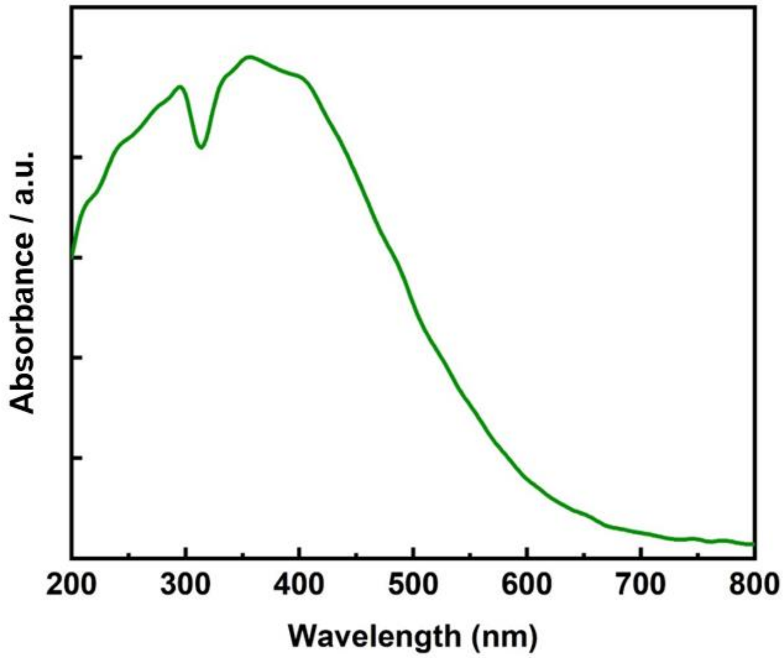

Figure S28. UV−vis absorption spectrum of solid DDC at room temperature.

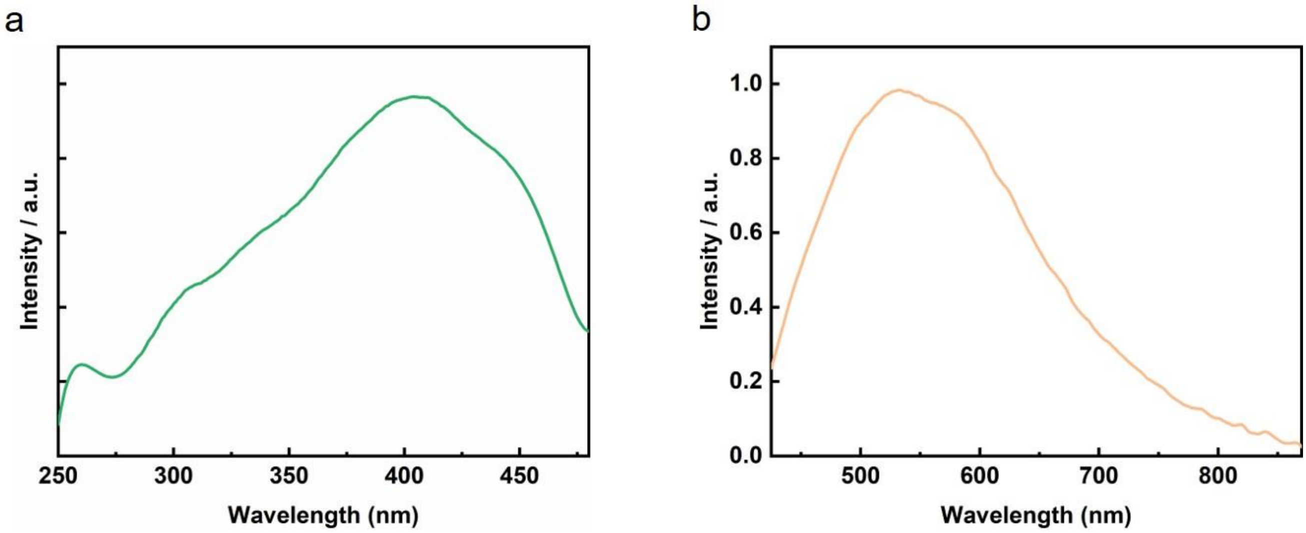

Figure S29. Excitation and emission spectra of solid DDC at room temperature. (a) Excitation spectrum of DDC crystals, showing the maximum excitation wavelength at 405 nm. (b) Emission spectrum of DDC crystals at an excitation source of 405 nm.

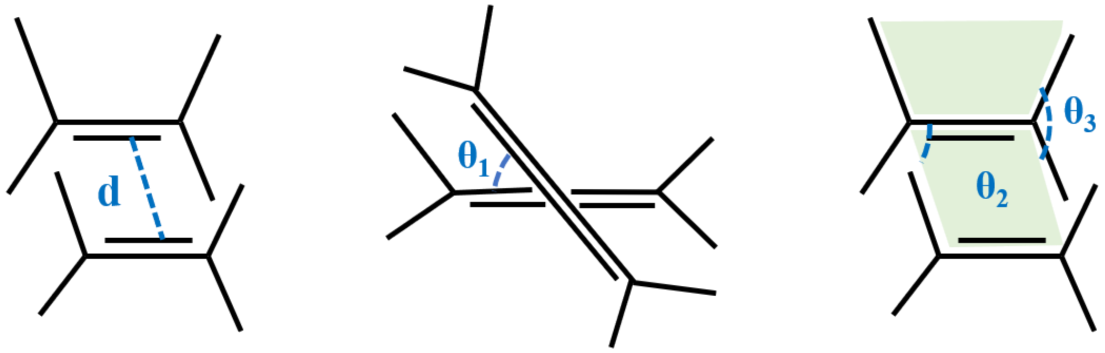

Figure S30. Definition of the geometric parameters considered to be geometric criteria relevant for the occurrence of the cycloaddition reaction.[17]

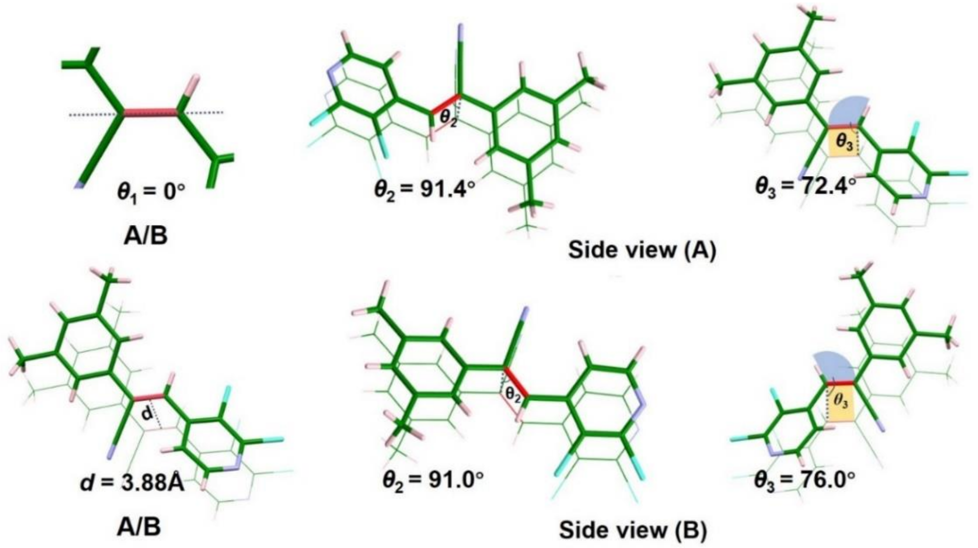

Figure S31. Inter-π-dimers in the structure of DPA.

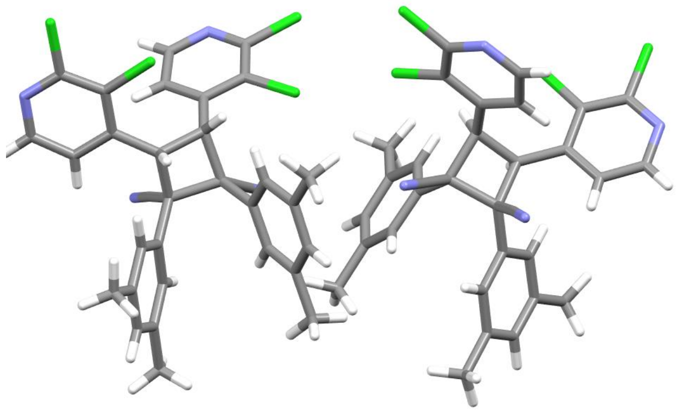

Figure S32. Molecular conformation of DDC molecules in the asymmetric unit. White atom— H, gray atom — C, blue atom— N, green atom— Cl.

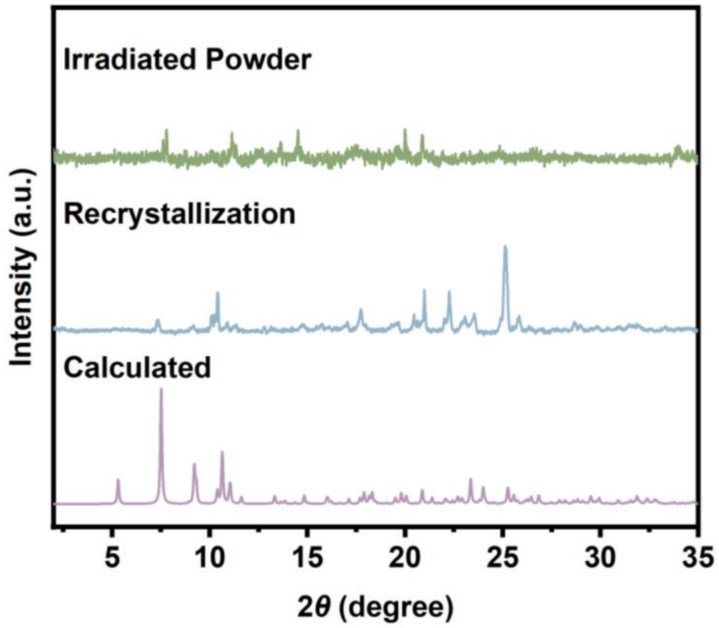

Figure S33. Comparison of the experimental powder diffraction patterns of irradiated powder, recrystallized sample, and the corresponding simulated pattern of the recrystallized sample.

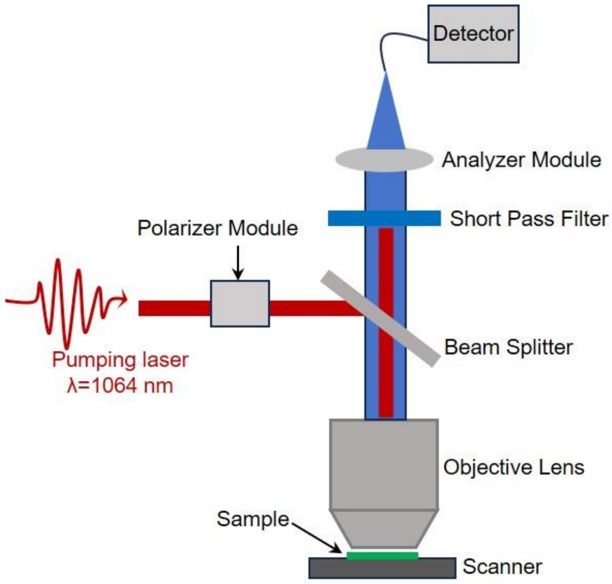

Figure S34. Schematic representation of the second-harmonic generation (SHG) test setup in the WITec alpha300 Raman system.

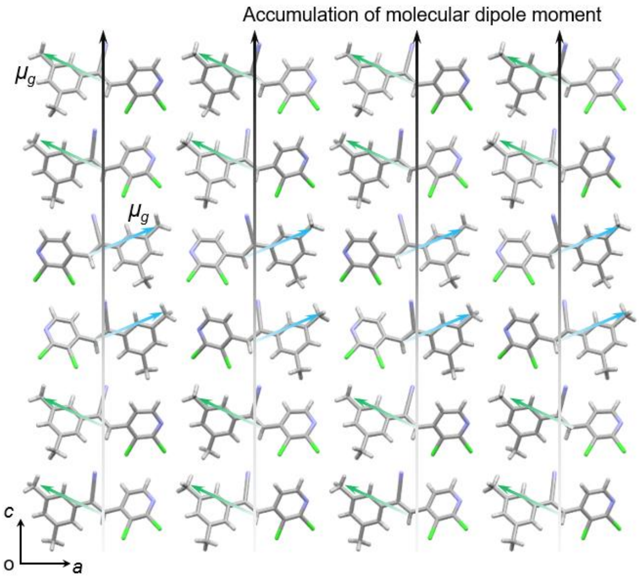

Figure S35. Depiction of the direction of the resultant dipole moment. μ_g is the ground state of the DPA molecule.

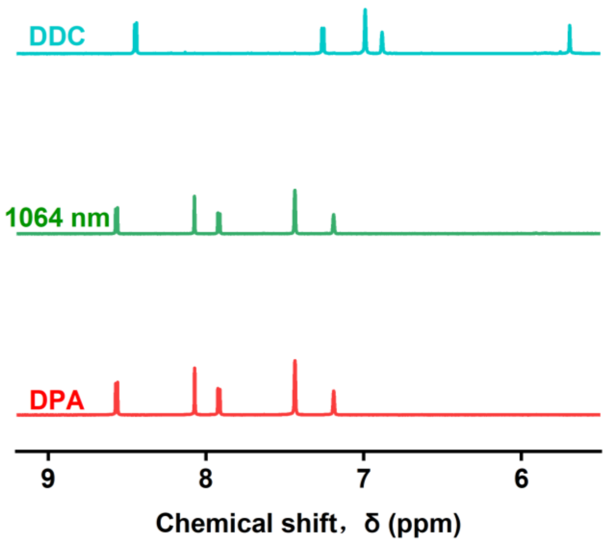

Figure S36. Characterization by ^{1}H NMR spectroscopy of the stability of DPA exposed to infrared radiation at 1064 nm for 12 h.

Table S1. Crystal structure data of DPA and DDC

	Straight DPA	Fiber DPA	Bent DPA	DDC
Crystal system	Orthorhombic	Orthorhombic	Orthorhombic	Monoclinic
Temperature / K	113	298	160	113
Space group	$Pca2_1$	$Pca2_1$	$Pca2_1$	$P2_1/c$
Z	8	8	8	8
Formula weight	303.18	303.18	303.18	606.37
Color	colorless	colorless	colorless	orange
a / Å	25.3067(5)	25.3939(3)	25.3158(6)	16.7285(5)
b / Å	3.88190(10)	3.97150(10)	3.89330(10)	23.4967(6)
c / Å	28.3415(6)	28.4457(5)	28.3161(7)	16.2061(4)
α / °	90	90	90	90
β / °	90	90	90	96.880(3)
γ / °	90	90	90	90
V / Å^3	2784.21(11)	2868.80(9)	2790.89(12)	6324.2(3)
Density/ (g/cm^3)	1.447	1.404	1.443	1.274
μ / mm^{-1}	0.456	3.980	4.091	0.401
λ (Mo/ Cu Kα) /Å	0.71073	1.54184	1.54184	0.71073
F_{000}	1248	1248	1248	2496
h_{min}, h_{max}	-31, 31	-30,30	-30, 26	-20, 20
k_{min}, k_{max}	-4, 3	-4, 2	-3, 4	-29, 29
l_{min}, l_{max}	-33, 35	-35, 34	-33, 33	-20, 20
No. of measured reflections	23033	14241	18138	54727
No. of unique reflections	5182	4596	4653	12921
No. of reflections used	4689	4384	4523	9265
No. of refinement parameters	365	365	364	729
R_{all}, R_{obs}	0.0496, 0.0431	0.0346, 0.0329	0.0537, 0.0530	0.0896, 0.0626
$wR_{2,all}$, $wR_{2,obs}$	0.1009, 0.0947	0.0925, 0.0908	0.1513, 0.1493	0.1616, 0.1440
$\Delta\rho_{min,max}$ / (e Å^{-3})	-0.268, 0.258	-0.239, 0.199	-0.516, 0.339	-0.550, 0.792
GooF	1.044	1.031	1.092	1.018
CCDC reference	2346259	2383608	2425281	2347873

Table S2. Elastic strain limit of the reported cyanostilbene derivatives

Compound	Plane	Strain limit/ %
(*Z*)−2-(3,5-bis(trifluoromethyl)phenyl)−3-(1-methyl-1H-imidazol-2-yl) acrylonitrile [18]	(001)	0.7
	(010)	2.8
(*Z*)-2-(3,5-bis(trifluoromethyl)phenyl)-3-(5-ethylthiophen-2-yl)acrylonitrile[19]	(010)	1.5-3.9
(*αZ*)-2-chloro-α-[(4-methoxyphenyl)methylene]-5-pyridinehyde[20]	(100)	4.6

Table S3. Modulus and hardness of straight DPA crystals based on nanoindentation

	Modulus/ GPa	Hardness/ GPa
1	10.717	0.292
2	10.735	0.295
3	11.928	0.319
4	11.71	0.266
5	11.217	0.301
6	11.468	0.290
7	11.527	0.307
8	12.013	0.320
9	11.591	0.312
10	12.033	0.313
11	10.702	0.297
12	11.21	0.305
13	12.122	0.322
14	11.87	0.354
15	11.874	0.323
16	11.979	0.310
17	11.341	0.328
18	11.373	0.330
19	11.937	0.334
20	11.413	0.354
	11.538 ± 0.452	0.314 ± 0.021

Table S4. Elastic modulus and hardness of the reported cyanostilbene derivatives

Compound	Plane	Elastic modulus / GPa	Hardness / GPa
(*Z*)−2-(3,5-bis(trifluoromethyl)phenyl)−3-(1-methyl-1H-imidazol-2-yl) acrylonitrile [18]	(001)	7.45 ± 0.18	0.11 ± 0.005
(*Z*)-2-(3,5-bis(trifluoromethyl)phenyl)-3-(5-methylthiophen-2-yl)acrylonitrile [19]	(010)	4.29 ± 0.20	0.12 ± 0.01
(*Z*)-2-(3,5-bis(trifluoromethyl)phenyl)-3-(5-ethylthiophen-2-yl)acrylonitrile[19]	(010)	6.91 ± 0.12	0.21 ± 0.01
(*Z*)-3-(4-Formylpheny)-2-(4-methoxyphenyl)acrylonitrile[21]	(010)	4.67	0.21
	(001)	16.65	0.17
(*αZ*)-2-chloro-α-[(4-methoxyphenyl)methylene]-5-pyridinehyde[20]	(100)	12.32	0.87

Table S5. Solvent Polarity and solvation free energy

Solvent	Polarity[22]	Solvation free energy kcal/mol
ethyl acetate	0.55	-980.6 ± 207.4
toluene	0.54	-813.4 ± 105.8
ethanol	0.54	-2128.6 ± 287.2
dichloromethane	0.82	

Legends to the supplementary movies

Movie S1: Growth-induced bending in ethyl acetate.

Movie S2: Growth-induced bending in ethyl acetate.

Movie S3: Growth-induced bending in a mixture of ethanol and dichloromethane.

Movie S4: Growth-induced bending in a mixture of ethanol and dichloromethane.

Movie S5: Mechanically induced bending on the (100) and (001) planes.

References

1. Sheldrick, G. M. A short history of SHELX. *Acta Crystallogr. A* **2008**, *64*, 112−122.

2. Sheldrick, G. M. SHELXT - Integrated space-group and crystal-structure determination. *Acta Crystallogr. A*, **2015**, *71*, 3−8.

3. Sheldrick, G. M. Crystal Structure Refinement with SHELXL. *Acta Crystallogr. C* **2015**, *71*, 3−8.

4. Dolomanov, O. V.; Bourhis, L. J.; Gildea, R. J.; Howard, J. A. K; Puschmann, H. OLEX2: A Complete Structure Solution, Refinement and Analysis Program. *J. Appl. Cryst.* **2009**, *42*, 339−341.

5. Oliver, W. C.; Pharr, G. M. An Improved Technique for Determining Hardness and Elastic Modulus using Load and Displacement Sensing Indentation Experiments. *J. Mater. Res.* **1992**, *7*, 1564–1583.

6. Oliver, W.C.; Pharr, G. M. Measurement of Hardness and Elastic Modulus by Instrumented Indentation: Advances in Understanding and Refinements to Methodology. *Mater. Res.* **2004**, *19*, 3–20.

7. Seth, S. K. Structural Elucidation and Contribution of Intermolecular Interactions in *O*-hydroxy acyl aromatics: Insights from X-ray and Hirshfeld Surface Analysis. *J. Mol. Struct.* **2014**, *1064*, 70–75.

8. Ling, I.; Alias, Y.; Sobolev, A. N.; Byrne, L. T.; Raston, C. L. Supramolecular Architecture Containing End-capping Bis-imidazolium cations. *CrystEngComm* **2011**, *13*, 787–793.

9. Lu, T.; Chen, Q. Interaction Region Indicator: a Simple Real Space Function Clearly Revealing both Chemical Bonds and Weak Interactions. *Chem.: Methods* **2021**, *1*, 231–239.

10. Johnson, E. R.; Keinan, S.; Mori-Sánchez, P.; Contreras-García, J.; Cohen, A. J.; Yang, W. T. Revealing Noncovalent Interactions. *J. Am. Chem. Soc.* **2012**, *132*, 6498–6506.

11. Lu, T.; Chen, F. Multiwfn: a Multifunctional Wavefunction Analyzer. *J. Comput. Chem.* **2012**, *33*, 580–592.

12. Humphrey, W.; Dalke, A.; Schulten, K. VMD: Visual Molecular Dynamics. *J. Mol. Graph.***1996,** *14*, 33–38.

13. Janert, P. K. Gnuplot in Action, 2nd Ed., **2016**.

14. Frisch, M. J.; Trucks, G. W.; Schlegel, H. B.; Scuseria, G. E.; Robb, M. A.; Cheeseman, J. R.; Scalmani, G.; Barone, V.; Petersson, G. A.; Nakatsuji, H.; Li, X.; Caricato, M.; Marenich, A. V.; Bloino, J.; Janesko, B. G.; Gomperts, R.; Mennucci, B.; Hratchian, H. P.; Ortiz, J. V.; Izmaylov, A. F.; Sonnenberg, J. L.; Williams; Ding, F.; Lipparini, F.; Egidi, F.; Goings, J.; Peng, B.; Petrone, A.; Henderson, T.; Ranasinghe, D.; Zakrzewski, V. G.; Gao, J.; Rega, N.; Zheng, G.; Liang, W.; Hada, M.;

Ehara, M.; Toyota, K.; Fukuda, R.; Hasegawa, J.; Ishida, M.; Nakajima, T.; Honda, Y.; Kitao, O.; Nakai, H.; Vreven, T.; Throssell, K.; Montgomery Jr., J. A.; Peralta, J. E.; Ogliaro, F.; Bearpark, M. J.; Heyd, J. J.; Brothers, E. N.; Kudin, K. N.; Staroverov, V. N.; Keith, T. A.; Kobayashi, R.; Normand, J.; Raghavachari, K.; Rendell, A. P.; Burant, J. C.; Iyengar, S. S.; Tomasi, J.; Cossi, M.; Millam, J. M.; Klene, M.; Adamo, C.; Cammi, R.; Ochterski, J. W.; Martin, R. L.; Morokuma, K.; Farkas, O.; Foresman, J. B.; Fox, D. J. Gaussian 16 Rev. C.01, Wallingford, CT, **2016**.

15. Dennington, R.; Keith, T. A.; Millam, J. M. GaussView, version 6.1; Semichem Inc., **2016.**

16. Kresse, G.; Furthmüller. Efficiency of ab-initio Total Energy Calculations for Metals and Semiconductors using a Plane-Wave Basis Set. *J. Phys. Rev. B* **1996**, *54*, 11169–11186

17. Yang, S.; Naumov, P.; Fukuzumi, S. Topochemical Limits for Solid-State Photoreactivity by Fine Tuning of the π-π Interactions. *J. Am. Chem. Soc.* **2009**, *131*, 7247–7249.

18. Lin, J.; Zhou, J.; Li, L.; Tahir, I.; Wu, S.; Naumov, P.; Gong, J. Highly Efficient *in crystallo* Energy Transduction of Light to Work. *Nat. Commun.* **2024**, *15*, 3633.

19. Lin, J.; Zhou, J.; Li, L.; Tahir, I.; Wu, S.; Naumov, P.; Gong, J. Distinct Mechanical Properties and Photomechanical Response from Isostructural, yet Chemically Different Molecular Crystal Actuators. *Chem. Mater.* **2024**, *36*, 8338–8348.

20. Yao, Y.; Wang, T.; Yu, H.; Jiang, Z.; Wu, W.; Zhao, H.; Wu, H.; Wang, N.; Huang, X.; Hao, H. Organic Polymorphic Crystals with Multi-stimuli Response: Excellent Mechanical Elasticity, Novel Two-stage Heterotropic Photochromism and Self-reversible Acidichromism. *Sci. China Mater.* **2024**, *67*, 2796–2806.

21. Jiang, Z.; Zhao, H.; Wu, W.; Chen, K.; Yu, H.; Wang, T.; Huang, X.; Wang, N.; Zhou, L.; Hao, H. Multi-stimuli Responsive Organic Polymorphic Crystals: Anisotropic Elasticity and Plasticity, Mechanochromism and Photomechanical Motions. *J. Mater. Chem. C* **2023**, *11*, 4375–4383.

22. Gu, C. H.; Li, H.; Gandhi, R. B.; Raghavan, K., Grouping Solvents by Statistical Analysis of Solvent Property Parameters: Implication to Polymorph Screening. *Int. J. Pharm.* **2004**, 283, 117–125.
